# Brazilian Guidelines for the pharmacological treatment of patients hospitalized with COVID-19 Joint guideline of Associação Brasileira de Medicina de Emergência, Associação de Medicina Intensiva Brasileira, Associação Médica Brasileira, Sociedade Brasileira de Angiologia e Cirurgia Vascular, Sociedade Brasileira de Infectologia, Sociedade Brasileira de Pneumologia e Tisiologia, Sociedade Brasileira de Reumatologia

**DOI:** 10.5935/0103-507X.20220001-en

**Published:** 2022

**Authors:** Maicon Falavigna, Cinara Stein, José Luiz Gomes do Amaral, Luciano Cesar Pontes de Azevedo, Karlyse Claudino Belli, Verônica Colpani, Clóvis Arns da Cunha, Felipe Dal-Pizzol, Maria Beatriz Souza Dias, Juliana Carvalho Ferreira, Ana Paula da Rocha Freitas, Débora Dalmas Gräf, Hélio Penna Guimarães, Suzana Margareth Ajeje Lobo, José Tadeu Monteiro, Michelle Silva Nunes, Maura Salaroli de Oliveira, Clementina Corah Lucas Prado, Vania Cristina Canuto Santos, Rosemeri Maurici da Silva, Marcone Lima Sobreira, Viviane Cordeiro Veiga, Ávila Teixeira Vidal, Ricardo Machado Xavier, Alexandre Prehn Zavascki, Flávia Ribeiro Machado, Carlos Roberto Ribeiro de Carvalho

**Affiliations:** 1 Institute for the Evaluation of Health Technology, Universidade Federal do Rio Grande do Sul - Porto Alegre (RS), Brazil.; 2 Hospital Moinhos de Vento - Porto Alegre (RS), Brazil.; 3 Associação Médica Brasileira - São Paulo (SP), Brazil.; 4 Associação de Medicina Intensiva Brasileira - São Paulo (SP), Brazil.; 5 Hospital das Clínicas, Faculdade de Medicina, Universidade de São Paulo - São Paulo (SP), Brazil.; 6 Sociedade Brasileira de Infectologia - São Paulo (SP), Brazil.; 7 Universidade Federal do Paraná - Curitiba (PR), Brazil.; 8 Experimental Pathophysiology Laboratory, Posgraduate Program in Health Sciences, Universidade do Extremo Sul Catarinense - Criciúma (SC), Brazil.; 9 Sociedade Brasileira de Pneumologia e Tisiologia - São Paulo (SP), Brazil.; 10 Heart Institute, Hospital das Clínicas, Faculdade de Medicina, Universidade de São Paulo - São Paulo (SP), Brazil.; 11 Associação Brasileira de Medicina de Emergência - São Paulo (SP), Brazil.; 12 Hospital de Pronto-Socorro de Porto Alegre - Porto Alegre (RS), Brazil.; 13 Faculdade de Medicina de São José do Rio Preto - São José do Rio Preto (SP), Brazil.; 14 Empresa Brasileira de Serviços Hospitalares - São Paulo (SP), Brazil.; 15 Department of Management and Incorporation of Technologies and Innovation in Health, Secretaria de Ciência, Tecnologia, Inovação e Insumos Estratégicos em Saúde, Ministério da Saúde - Brasília (DF), Brazil.; 16 Sociedade Brasileira de Angiologia e Cirurgia Vascular - São Paulo (SP), Brazil.; 17 Hospital das Clínicas, Faculdade de Medicina de Botucatu, Universidade Estadual Paulista - Botucatu (SP), Brazil.; 18 BP - A Beneficência Portuguesa de São Paulo - São Paulo (SP), Brazil.; 19 Sociedade Brasileira de Reumatologia - São Paulo (SP), Brazil.; 20 Hospital São Paulo, Escola Paulista de Medicina, Universidade Federal de São Paulo - São Paulo (SP), Brazil.

**Keywords:** COVID-19, COVID-19/ drug therapy, Health planning guidelines, SARS-CoV-2, Brazil

## Abstract

**Objective:**

Several therapies are being used or proposed for COVID-19, and many lack appropriate evaluations of their effectiveness and safety. The purpose of this document is to develop recommendations to support decisions regarding the pharmacological treatment of patients hospitalized with COVID-19 in Brazil.

**Methods:**

A group of 27 experts, including representatives of the Ministry of Health and methodologists, created this guideline. The method used for the rapid development of guidelines was based on the adoption and/or adaptation of existing international guidelines (GRADE ADOLOPMENT) and supported by the e-COVID-19 RecMap platform. The quality of the evidence and the preparation of the recommendations followed the GRADE method.

**Results:**

Sixteen recommendations were generated. They include strong recommendations for the use of corticosteroids in patients using supplemental oxygen, the use of anticoagulants at prophylactic doses to prevent thromboembolism and the nonuse of antibiotics in patients without suspected bacterial infection. It was not possible to make a recommendation regarding the use of tocilizumab in patients hospitalized with COVID-19 using oxygen due to uncertainties regarding the availability of and access to the drug. Strong recommendations against the use of hydroxychloroquine, convalescent plasma, colchicine, lopinavir + ritonavir and antibiotics in patients without suspected bacterial infection and also conditional recommendations against the use of casirivimab + imdevimab, ivermectin and rendesivir were made.

**Conclusion:**

To date, few therapies have proven effective in the treatment of hospitalized patients with COVID-19, and only corticosteroids and prophylaxis for thromboembolism are recommended. Several drugs were considered ineffective and should not be used to provide the best treatment according to the principles of evidence-based medicine and promote economical resource use.

## INTRODUCTION

COVID-19, the disease caused by severe acute respiratory syndrome coronavirus 2 (SARS-CoV-2), was first identified in Wuhan, China, in December 2019.^(1)^ With the global escalation of new cases, on January 30, 2020, the World Health Organization (WHO) decreed the outbreak of the new coronavirus a public health emergency of international interest; in March 11, 2020, the WHO designated it as a pandemic.^(2)^ Since then, COVID-19 has become a matter of global concern that requires global efforts for its prevention and control.

Worldwide, as of October 10, 2021, the WHO had reported more than 237.5 million confirmed cases and more than 4.8 million deaths due to COVID-19.^(3)^ In Brazil, as of October 15, 2021, 21,612,237 COVID-19 cases and 602,099 deaths due to COVID-19 were confirmed.^(4)^ In most cases, people with COVID-19 have a mild clinical presentation of the disease, with symptoms such as fever, dry cough and fatigue, and the disease resolves in a self-limited manner. However, approximately 14% of COVID-19 patients develop severe disease, which may require oxygen therapy or hospitalization, and 5% require care in the intensive care unit (ICU).^(5)^ Patients with COVID-19 who require ICU admission for acute respiratory failure due to viral pneumonia usually exhibit an increased respiratory rate and hypoxemia, which may progress to sepsis, septic shock and multiple organ failure, including acute kidney injury and cardiac injury.^(6)^

In the context of a pandemic, most actions and interventions are empirical and based on findings that are often derived only from *in vitro* experiments, anecdotal personal experiences and small and methodologically limited observational studies. There is an incessant and often uncoordinated search for treatments, and drugs with doubtful effectiveness are quickly proclaimed as potentially lifesaving and included in treatment protocols. The clinical decision-making process, which is usually guided by a rational, evidence-based approach, becomes clearly emotional. Although this may be understandable from a humanitarian and social point of view in a pandemic context, this process can lead to excess secondary treatment and uses without indication, with consequent risks of adverse events.^(7-9)^ In contexts such as the current one, the development of guidelines based on the best available evidence is useful to guide health professionals in decisionmaking.

This guideline for the pharmacological treatment of patients hospitalized with COVID-19 was developed by the Ministry of Health in conjunction with seven medical specialty societies. The objective of the document was to provide uniformity in the therapeutic indications for patients with COVID-19 in the context of hospital treatment and to guide therapeutic interventions, making use of the best evidence available at the time of its elaboration.

## METHODS

This guideline followed the method for developing rapid guidelines based on the adoption and/or adaptation of recommendations in existing international guidelines, which were identified through the e-COVID-19 RecMap platform and additional searches, and the addition of new recommendations when necessary (GRADE ADOLOPMENT).^(10,11)^ The target audience was composed of health professionals involved in the care of adult patients hospitalized with COVID-19, especially intensivists, internists, emergency physicians, infectious disease specialists, pulmonologists and clinical pharmacists.

### Guideline development group

The group involved in the development of this guideline was composed of a panel of experts under the management of the Department of Management and Incorporation of Technologies and Innovation in Health (DGITIS - *Departamento de Gestão e Incorporação de Tecnologias e Inovação em Saúde*) of the Secretariat of Science, Technology and Strategic Inputs (SCTIE - *Secretaria de Ciência, Tecnologia e Insumos Estratégicos*) of the Ministry of Health. The panel of experts included intensive care physicians, internists and emergency physicians, vascular and endovascular surgeons, infectious disease specialists, rheumatologists, pulmonologists, pharmacists, representatives of the Ministry of Health and methodologists. The following medical societies participated in the development of this guideline and endorsed its recommendations: *Associação Brasileira de Medicina de Emergência* (ABRAMEDE), *Associação de Medicina Intensiva Brasileira* (AMIB), *Associação Médica Brasileira* (AMB), *Sociedade Brasileira de Angiologia e Cirurgia Vascular* (SBACV); *Sociedade Brasileira de Infectologia* (SBI), *Sociedade Brasileira de Pneumologia e Tisiologia* (SBPT), *Sociedade Brasileira de Reumatologia* (SBR).

Between the end of March and the beginning of May 2021, the management committee organized seven virtual meetings with the experts by videoconference to present the identified international guidelines and recommendations, discuss the evidence with the experts and develop guidelines adapted to the national context. The members of the management committee and the methodologists did not interfere in the experts’ preparation of the guidelines. The list of participants, their role in the guideline and the declaration of conflicts of interest are presented in the Supplementary Material.

This guideline was presented to the National Commission for the Incorporation of Technologies into the SUS (Conitec) on May 13, 2021, are it was evaluated in a public consultation process, and its final version, with the recommendations presented here, was approved on June 10, 2021. Conitec is a committee composed of 13 members representing the Ministry of Health, health councils and regulatory agencies. The original guideline was published on the Conitec website and in the Brazilian Federal Official Gazette, and the article presented here is a document for dissemination.

In October 2021, the recommendation regarding the use of anticoagulants was updated due to newly published relevant evidence.

### Research questions

To identify the clinical issues of interest, the technologies evaluated in other national and international guidelines for the treatment of COVID-19 were reviewed. Twelve clinical questions were prepared according to the PICO method (population, intervention, comparator and outcome) to consider the following therapies: anticoagulants, antimicrobials, azithromycin, casirivimab + imdevimab, colchicine, corticosteroids, hydroxychloroquine, ivermectin, lopinavir/ritonavir, convalescent plasma, rendesivir and tocilizumab. Each research question could generate one or more recommendations. The research questions that were addressed are listed in the Supplementary Material.

### Search and synthesis of evidence

The source documents for the identification of evidence were existing guidelines; systematic reviews were not conducted for the developed issues. The recommendations, evidence profiles, and Grading of Recommendations Assessment, Development and Evaluation (GRADE) domains were extracted from the evidence tables for decision-making using the e-COVID-19 RecMap platform. The original documents were evaluated when necessary.^(10,11)^ The following guidelines were used in the adaptation process:

- World Health Organization (WHO): Therapeutics and COVID-19 - Living Guideline (March 2021).^(6)^
- A ustralian National COVID-19 Clinical Evidence Taskforce: Caring for People with COVID-19. Supporting Australia’s Healthcare Professionals with Continually Updated, Evidence-Based Clinical Guidelines (April 2021).^(12)^
- Infectious Diseases Society of America (IDSA): Infectious Diseases Society of America Guidelines on the Treatment and Management of Patients with COVID-19 (April 2021).^(13)^
- A MIB, SBI and SBPT: *Diretrizes para o tratamento farmacológico da COVID-19. Consenso da Associação de Medicina Intensiva Brasileira, da Sociedade Brasileira de Infectologia e da Sociedade Brasileira de Pneumologia e Tisiologia* (June 2020).^(14)^
- National Institute for Health and Care Excellence (NICE): COVID-19 Rapid Guideline: Managing COVID-19 (March 2021).^(15)^
- National Institutes of Health (NIH): Coronavirus Disease 2019 (COVID-19) Treatment Guidelines (April 2021).^(16)^
- Surviving Sepsis Campaign (SSC): Society of Critical Care Medicine/European Society of Intensive Care Medicine Surviving Sepsis Campaign Guidelines on the Management of Adults with Coronavirus Disease 2019 (COVID-19) in the ICU: First Update (March 2021).^(17)^
- European Respiratory Society (ERS): Management of Hospitalized Adults with Coronavirus Disease 2019 (COVID-19): a European Respiratory Society Living Guideline (April 2021).^(18)^
- A merican Society of Hematology (ASH): American Society of Hematology 2021 Guidelines on the Use of Anticoagulation *for Thromboprophylaxis in Patients with COVID-19* (October 2020).^(19)^
- European League against Rheumatism (EULAR): EULAR Points to Consider on Pathophysiology and the Use of Immunomodulatory Therapies in COVID-19 (January 2021).^(20)^

### Assessment of the certainty of evidence and the development of recommendations

To evaluate the certainty of the evidence, the GRADE system was used. We adopted the GRADE evidence profiles presented by the guideline that most recently conducted an evidence search that answered the research questions of interest. When it was necessary to update information, a structured literature search was performed, including preprints and press releases regarding studies by research groups (COALIZÃO, RECOVERY, REMAP-CAP and SOLIDARITY) when appropriate. Evidence from preprints and press releases was considered a qualitative factor in decision-making and did not modify the level of evidence evaluated by the original documents (Table 1).

**Table 1:** Certainty of evidence according to the GRADE system

Level	Definition	Implications
High	Strong confidence that the true effect lies close to that of the effect estimate	It is unlikely that additional trial will change the confidence in the estimation effect
Moderate	Moderate confidence in the effect estimated	Future trial may modify the confidence in the effect estimate, and also can change the estimate
Low	Limited confidence in the effect estimate.	Future trials are likely to important impact on our confidence in the estimated effect
Very low	Uncertain confidence in the effect estimate.	Any estimate of effect is uncertain

Source: Schünemann H, Brozek J, Guyatt G, Oxman A, editors. GRADE Handbook. Handbook for grading the quality of evidence and the strength of recommendations using the GRADE approach. Chapter 5, Table 5.1. Quality of evidence grades. [Updated October 2013]. [cited 2021 June 10]. Available from: https://gdt.gradepro.org/app/handbook/handbook.html.^(21)^

According to the GRADE methodology, recommendations can be strong or conditional (weak) for or against an intervention. The strength of the recommendations is shown in table 2.

**Table 2:** Strength of recommendation according to the GRADE system

Target audience	Strong	Conditional (weak)
Policymakers	The recommendation should be adopted as a healthcare policy in most of the situations	Substantial debate required and the involvement of stakeholders
Clinicians	Most individuals would want the intervention to be indicated, and only a small number would reject this recommendation	A large portion of the individuals would want the intervention to be indicated; however, some individuals would reject this recommendation
Patients	Most of the patients should receive the recommended intervention	The clinician should acknowledge that different choices are appropriate for each patient and choose consistently with his/her values and preferences

Source: Schünemann H, Brozek J, Guyatt G, Oxman A, editors. GRADE Handbook. Handbook for grading the quality of evidence and the strength of recommendations using the GRADE approach. Chapter 6, Table 6.1. Implications of strong and weak recommendations for different users of guidelines. [Updated October 2013]. [cited 2021 June 10]. Available from: https://gdt.gradepro.org/app/handbook/handbook.html.^(21)^

In developing the recommendations, the evidence of benefits and risks, the certainty of evidence, the costs and use of resources, the acceptance by professionals and other barriers to implementation were considered. Additional statements about the recommendations, such as potential exceptions to the proposed behaviors or clarifications of them, are documented throughout the text. The direction and strength of the recommendations, as well as their wording, were determined during the meetings at which the recommendations were prepared.

### Population of interest

The target population of the recommendations is adult hospitalized patients with a diagnosis or suspicion of COVID-19. Nonhospitalized patients with COVID-19 and pregnant and postpartum women were not targets of this guideline.

## RESULTS

**Figure 1:**
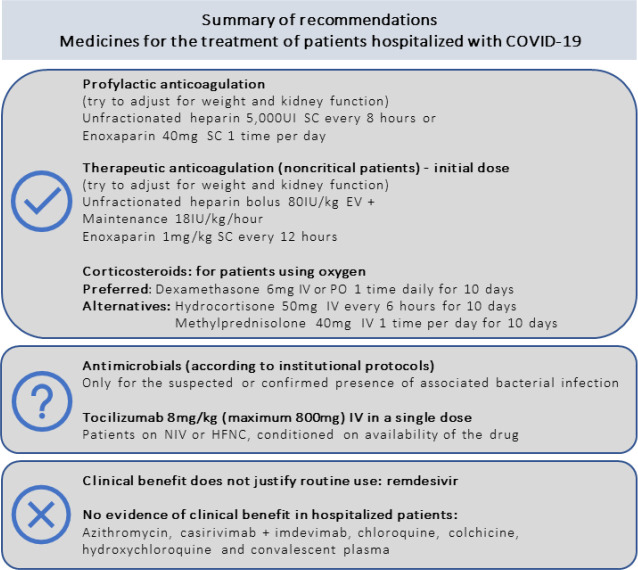
Recommendations for pharmacological treatment of patients hospitalized with COVID-19. SC - subcutaneous; IV - intravenously; PO - orally; NIV - noninvasive ventilation; HFNC - high-flow nasal cannula.

Sixteen recommendations were made. The recommendations are summarized in table 3 and in figure 1.

**Table 3:** Summary of recommendations

Medication	Recommendation
**Corticosteroids**	**Recommendation 1.1 -** We recommend the use of 6 mg dexamethasone intravenously or orally once daily for 10 days in patients who are hospitalized with COVID-19 and using supplemental oxygen (strong recommendation, moderate certainty of evidence) **Recommendation 1.2 -** We suggest against the use corticosteroids in patients hospitalized with COVID-19 who are not using supplemental oxygen (conditional recommendation, low certainty of evidence)
**Anticoagulants**	**Recommendation 2.1 -** We recommend the use of anticoagulants at prophylactic doses for VTE in critically ill patients (those using vasoactive drugs and those undergoing renal replacement therapy, HFNC, NIV or IMV) with COVID-19 (nongraded recommendation) **Recommendation 2.2 -** We suggest against the use intermediate doses or therapeutic anticoagulation in critically ill COVID-19 patients (those using vasoactive drugs or undergoing renal replacement therapy, HFNC, NIV or IMV) without evidence of thromboembolism (conditional recommendation, very low certainty of evidence) **Recommendation 2.3 -** We suggest the use heparin or enoxaparin in therapeutic doses in noncritical patients (those with no need for vasoactive drugs, renal replacement therapy, HFNC, NIV or IMV) hospitalized with COVID-19 (conditional recommendation, very low certainty of evidence)
**Antimicrobials**	**Recommendation 3.1 -** We recommend against the use antimicrobials in patients with COVID-19 without suspected bacterial infection (nongraded recommendation)
**Tocilizumab**	**Recommendation 4.1 -** The use of tocilizumab is clinically indicated for hospitalized patients with COVID-19 using NIV or HFNC; however, it is not possible to recommend it at this time (May 2021), as this use is not indicated in the label and there are uncertainties regarding access to the drug that affect the ability to meet the potential demand (no recommendation, moderate certainty of evidence) **Recommendation 4.2 -** We suggest against the use tocilizumab in patients on mechanical ventilation (conditional recommendation, moderate certainty of evidence)
**Chloroquine or hydroxychloroquine**	**Recommendation 5.1 -** We recommend against the use chloroquine or hydroxychloroquine in patients hospitalized with COVID-19 (strong recommendation, moderate certainty of evidence)
**Azithromycin**	**Recommendation 6.1 -** We recommend against the use azithromycin, with or without chloroquine or hydroxychloroquine, in patients hospitalized with COVID-19 (strong recommendation, moderate certainty of evidence)
**Casirivimab + imdevimab**	**Recommendation 7.1 -** We suggest against the use casirivimab + imdevimab in patients hospitalized with COVID-19 (conditional recommendation, very low certainty of evidence)
**Rendesivir**	**Recommendation 8.1 -** We suggest against the use rendesivir in patients hospitalized with COVID-19 (conditional recommendation, low certainty of evidence)
**Convalescent plasma**	**Recommendation 9.1 -** We recommend against the use convalescent plasma in patients hospitalized with COVID-19 (strong recommendation, moderate certainty of evidence)
**Ivermectin**	**Recommendation 10.1 -** We suggest against the use ivermectin in patients hospitalized with COVID-19 (conditional recommendation, very low certainty of evidence)
**Colchicine**	**Recommendation 11.1 -** We recommend against the use colchicine in patients hospitalized with COVID-19 (strong recommendation, moderate certainty of evidence)
**Lopinavir/ritonavir**	**Recommendation 12.1 -** We recommend against the use lopinavir/ritonavir in patients hospitalized with COVID-19 (strong recommendation, moderate certainty of evidence)

VTE - venous thromboembolism; HFNC - high-flow nasal cannula; NIV - noninvasive ventilation; IMV - invasive mechanical ventilation.

Below, we present the recommendations, the rationale for the decision and, when relevant, considerations for implementation. Detailed information on the evidence supporting each recommendation is presented in the Supplementary Material.

### Corticosteroids

**Recommendation 1.1 -** We recommend the use of 6mg dexamethasone intravenously (IV) or orally (PO) once daily for 10 days in patients hospitalized with COVID-19 using supplemental oxygen (strong recommendation, moderate certainty of evidence).

**Recommendation 1.2 -** We suggest against the use corticosteroids in patients hospitalized with COVID-19 who are not using supplemental oxygen (conditional recommendation, low certainty of evidence).

**Justification for the recommendation -** The panel of experts considered that there is an important benefit gained from the use of corticosteroids in patients hospitalized with COVID-19 who are using oxygen.^(22,23)^ Along with the proven benefit, which has a moderate certainty of evidence, the drug is well tolerated, widely available and inexpensive, which leads to a strong recommendation in favor of its use in this population. The available evidence suggests a lack of benefit in patients who do not require supplemental oxygen.

**General and implementation considerations -** The preferred drug for use is dexamethasone, as used in the RECOVERY study.^(23)^ Alternatively, if dexamethasone is not available, hydrocortisone can be used at a dose of 50mg IV every 6 hours, or methylprednisolone can be used at a dose of 40mg IV per day. Other corticosteroids can be used at equivalent doses, such as prednisone 40mg once a day PO. Oral corticosteroids should be used only in patients with a patent enteral route and may be administered with food. If there is no certainty regarding the suitability of the enteral route (e.g., in a critically ill patient), IV should be used whenever possible.

The use of corticosteroids as recommended (at low doses, limited to 10 days) may be abruptly discontinued, and gradual withdrawal is not necessary. There is also no need to continue treatment after discharge. There is uncertainty regarding the optimal dose for patients on mechanical ventilation (MV), and higher doses, limited to 20mg per day of dexamethasone or 100mg per day of methylprednisolone, may be used.^(22,24)^ It is not possible to make recommendations regarding the replacement of dexamethasone with hydrocortisone in patients with COVID-19 and septic shock, as both alternatives are valid at the established doses; however, the two should not be used concomitantly. There is no evidence of benefit for the use of corticosteroid pulse therapy in patients with COVID-19; the effects of immunosuppression on disease progression are not known, and an increased risk of associated infections is expected.

Patients with other indications for corticosteroids (for example: exacerbated asthma or chronic obstructive pulmonary disease, previous use due to rheumatic diseases, pulmonary maturation in pregnant women) should receive them so according to their clinical indication.

### Anticoagulants

**Recommendation 2.1 -** We recommend the use of anticoagulants at prophylactic doses for venous thromboembolism (VTE) in critically ill patients (those using vasoactive drugs or receiving renal replacement therapy, high-flow nasal cannula (HFNC), noninvasive ventilation (NIV) or invasive mechanical ventilation (IMV)) with COVID-19 (nongraded recommendation).

**Recommendation 2.2 -** We suggest against the use intermediate doses or therapeutic anticoagulation in critically ill patients (those using vasoactive drugs or undergoing renal replacement therapy, HFNC, NIV or IMV) with COVID-19 without evidence of thromboembolism (conditional recommendation, very low certainty of evidence).

**Recommendation 2.3 -** We suggest the use heparin or enoxaparin in therapeutic doses in noncritical patients (those who do not need vasoactive drugs, renal replacement therapy, HFNC, NIV or IMV) hospitalized with COVID-19 (conditional recommendation, very low certainty of evidence).

**Justification for the recommendation -** The panel of experts considered that there is no benefit from the use of anticoagulants at intermediate or therapeutic doses in critically ill patients with COVID-19. Additionally, anticoagulation is associated with an increased risk of bleeding events and should be avoided in this population. There is a potential benefit from the use of heparin or enoxaparin at therapeutic doses in noncritical patients, and the same effect was not observed for oral anticoagulants.

**General considerations and considerations for implementation -** In noncritical hospitalized patients (i.e., those who do not need vasoactive drugs, renal replacement therapy, HFNC, NIV or IMV), therapeutic anticoagulation with unfractionated heparin or enoxaparin may be used according to the individual’s risk of bleeding. Oral anticoagulants are not effective in this population and should not be used for this purpose. Rivaroxaban is not effective in the treatment of hospitalized patients with COVID-19 and is associated with a greater number of potential adverse events.^(25)^

Prophylaxis for VTE should be performed, preferably with unfractionated heparin, although enoxaparin or fondaparinux may be used alternatively. The suggested dosage is shown in table 4. The preference for unfractionated heparin over enoxaparin is based on lower costs and greater availability of the former at the time the recommendation was drafted; however, availability may vary over time and among institutions.

**Table 4:** Dosage of anticoagulant drugs

Patient group/medication	Dose
**Noncritical patients: therapeutic anticoagulation**	
Unfractionated heparin Standard dose	Start with a bolus of 80IU/kg IV + maintenance: 18IU/kg/hour Adjust according to the coagulogram, maintaining the RT between 1.5 and 2.5, according to Raschke et al.^(29)^
Enoxaparin If ClCr > 30mL/minute If ClCr < 30mL/minute	1 mg/kg subcutaneously every 12 hours. Attention to weight extremes: If weight is < 40kg or > 150kg, it is suggested to monitor anti-Xa activity (0.3 - 0.7)^(30,31)^ or If BMI is > 40kg/m^2^: 0.7 - 0.8mg/kg. Consider a limit of 150mg/dose^(30)^ Avoid. Instead, unfractionated heparin, controlled according to the coagulogram (RT of 1.5 - 2.3), is suggested^(29)^
Fondaparinux If ClCr > 20mL/minute If ClCr < 20mL/minute	< 50kg: 5mg subcutaneously, once daily 50 - 100kg: 7.5mg subcutaneously, once daily > 100kg:10mg subcutaneously, once daily *Between 20 - 30mL/minute, consider administering one dose every 48 hours Do not use
**Critically ill patients: prophylaxis**	
Unfractionated heparin Standard dose Patients with BMI > 40kg Renal insufficiency (ClCr < 30mL/minute)	5,000IU subcutaneously every 8 hours 10,000IU every 12 hours 5,000IU every 12 hours
Enoxaparin Up to 80kg Between 80 and 120kg Over 120kg BMI > 50kg ClCr < 30mL/minute	40mg once a day 60mg once a day 40mg every 12 hours 60mg every 12 hours Do not use
Fondaparinux* Standard dose Renal insufficiency (ClCr 20 to 30mL/minute) Renal insufficiency (ClCr < 20mL/minute)	2.5mg once a day 2.5mg every 48 hours Do not use

IV - intravenous ; RT - ratio of the activated partial thromboplastin time; ClCr - creatinine clearance; BMI - body mass index; IU - international units.

* Avoid fondaparinux in patients weighing less than 50kg due to the increased risk of bleeding.

The definition of preferential alternatives can be customized based on the particularities of each institution. Enoxaparin and fondaparinux appear to have similar results; however, enoxaparin has the advantage of a greater number of studies and more experience with its use.

Fondaparinux is indicated in patients with suspected or diagnosed heparin-induced thrombocytopenia and may also be used preferentially in patients with thrombocytopenia due to other etiologies. Prophylaxis is contraindicated in patients with platelet counts < 30,000 platelets per mm^3^.

There is no indication for the routine use of anticoagulants postdischarge for COVID-19. The indication for the use of anticoagulants after discharge should follow the same criteria applied for non-COVID-19 patients according to institutional protocols, and instruments such as the Padua score and IMPROVE may be used as support.^(26-28)^ Anticoagulation therapy should be used for patients with specific clinical indications (e.g., atrial fibrillation and VTE) according to their baseline condition.

### Antimicrobials

**Recommendation 3.1 -** We recommend against the use antimicrobials in patients with COVID-19 without suspected bacterial infection (nongraded recommendation).

**Justification for the recommendation -** The panel of experts determined that there is no basis for the routine use of antimicrobials in patients with COVID-19 without suspected associated bacterial infection, since coinfection is uncommon.^(32)^

**General considerations and for implementation -** Patients with suspected sepsis on admission who do not have a definitive diagnosis of COVID-19 should be managed according to the institutional protocol for sepsis.

Patients with COVID-19 who, on hospital admission, have a potential bacterial focus of infection (e.g., pulmonary radiological consolidation, leukocytosis in the absence of corticosteroid use, purulent secretions) are potential candidates for the empirical use of antimicrobials. The initiation of antimicrobial use should be based on clinical judgment, patient risk factors and local epidemiology. Bacterial cultures (blood culture and culture of the site of suspicion) should be collected prior to the initiation of antimicrobials. Empirical therapy should be based on guidelines from the local Hospital Infection Control Service and/or institutional protocols for the use of antimicrobials. Daily reassessments should be performed to determine the need for de-escalation or suspension of antimicrobial therapy.

A high level of suspicion of health care-related infections, such as MV-associated pneumonia, urinary tract infection, and catheter-associated bloodstream infection, should be maintained.

### Tocilizumab

**Recommendation 4.1-** The use of tocilizumab is clinically indicated in hospitalized patients with COVID-19 using NIV or HFNC; however, it is not possible to recommend it at this time (May 2021) as this use is off label and there are uncertainties regarding access to this drug due to limited ability to meet the potential demand (no recommendation, moderate certainty of evidence).

**Recommendation 4.2 -** We suggest against the use tocilizumab in patients on MV (conditional recommendation, moderate certainty of evidence).

**Justification for the recommendation -** The expert panel understands that tocilizumab is beneficial for patients who are hospitalized with COVID-19 and using supplemental oxygen who are not on MV.^(33,34)^ However, it is not possible to routinely recommend it, as there is not an adequate supply of the drug for the population that could potentially benefit from it. Given the limited availability of the drug, if it is used, it should be offered to patients with recent clinical deterioration, the onset of NIV or HFNC in the last 24 hours and the risk of progression to MV. There is no defined benefit of tocilizumab for hospitalized patients on MV. Thus, in the current context, its use in this population is not recommended.

Although the evidence points to a benefit, it is important to note that use of this immunomodulator for patients with COVID-19 is not indicated in the label as it has not been evaluated by the National Health Surveillance Agency (Anvisa - *Agência Nacional de Vigilância Sanitária*), which is currently the holder of the drug. The registry did not request an expansion of the drug’s use. In this sense, the manufacturer of the product itself warned of excess demand that could harm patients for whom the medication has an established indication, especially those with severe rheumatoid arthritis, if the drug becomes unavailable because of its prescription for COVID-19.^(35,36)^ This recommendation should be reviewed as soon as there is greater availability of tocilizumab.

**General and implementation considerations -** Currently, if tocilizumab is available, patients who have the greatest potential to benefit from its use should be prioritized. According to clinical judgment, patients with recent clinical deterioration should be prioritized, including those with the onset of NIV or HFNC use in the last 24 hours and risk of progression to MV. Evidence suggests that the benefit of tocilizumab is dependent on the coadministration of corticosteroids.^(33)^ Tocilizumab should be preferentially used for patients with increased inflammatory markers, such as C-reactive protein, ferritin and lactic dehydrogenase, since this is the population most frequently evaluated in clinical studies. Although studies show that tocilizumab is beneficial for patients using low-flow oxygen, this group should not be prioritized. These patients should be monitored, and if they experience clinical deterioration requiring NIV or HFNC, they become a priority group for the use of this medication. Tocilizumab should be used at a single dose of 8mg/kg IV, with a maximum dose of 800mg. A second dose of tocilizumab should not be administered until the supply of the drug is stabilized. If tocilizumab is used, it should always be accompanied by corticosteroids, and dexamethasone 6mg IV or PO is the recommended regimen.

Attention should be paid to the presence of latent infections, such as tuberculosis and parasitic infections, for which tocilizumab can promote reactivation, especially in critically ill patients already using corticosteroids. Tocilizumab should not be used in patients with the presence or suspicion of associated bacterial infections. It should be used with caution in immunosuppressed patients. The drug should not be used in patients with neutropenia (< 500 cells), thrombocytopenia (< 50,000) or transaminase levels five times above the normal range. Tocilizumab should be preferred in hospitals that have experience with its use and with the management of its potential adverse events.

### Chloroquine, hydroxychloroquine and azithromycin

**Recommendation 5.1 -** We recommend against the use chloroquine or hydroxychloroquine in patients hospitalized with COVID-19 (strong recommendation, moderate certainty of evidence).

**Recommendation 6.1 -** We recommend against the use azithromycin, with or without chloroquine or hydroxychloroquine, in patients hospitalized with COVID-19 (strong recommendation, moderate certainty of evidence).

**Justification for the recommendation -** The panel of experts considered that the evidence shows no benefit from the use of hydroxychloroquine, chloroquine or azithromycin in patients hospitalized with COVID-19.^(37-44)^ The drugs were not recommended by any of the identified guidelines.

**General and implementation considerations -** Chloroquine and hydroxychloroquine should not be used, regardless of the route of administration (oral, inhaled or other). Patients who use chloroquine or hydroxychloroquine due to other health conditions (e.g., rheumatic diseases and malaria) should continue to use them.

Azithromycin can be used in cases of suspected or confirmed bacterial infection, according to the guidelines of the local Hospital Infection Control Service and/or institutional protocols for the use of antimicrobials.

### Casirivimab + imdevimab

**Recommendation 7.1 -** We suggest against the use casirivimab + imdevimab in patients hospitalized with COVID-19 (conditional recommendation, very low certainty of evidence).

**Justification for the recommendation -** The expert panel considered that despite presenting promising results in patients in the early stages of the disease,^(45,46)^ there is no evidence to support the use of casirivimab + imdevimab in hospitalized patients, and the inclusion of patients in clinical studies should be encouraged.

**General considerations and considerations for implementation -** In addition to casirivimab + imdevimab, other monoclonal antibodies (bamlanivimab and etesevimab) are being studied for use in COVID-19 but have no documented benefit in this population. The inclusion of hospitalized patients in clinical studies of these drugs is encouraged.

### Rendesivir

**Recommendation 8.1 -** We suggest against the use rendesivir in patients hospitalized with COVID-19 (conditional recommendation, low certainty of evidence).

**Justification for the recommendation -** Although the ACTT-1 study showed reduced progression to MV and reduced mortality in patients using low-flow oxygen, no reduction in mortality was observed in the SOLIDARITY study, which had a larger number of patients. The study group considered that there is uncertainty regarding the benefit of using rendesivir, but there is no justification for its routine use in patients hospitalized with COVID-19.^(47-49)^ These uncertainties regarding the clinical benefit, along with the high cost, low availability and low experience with the use of this drug, justify the conditional recommendation against the use of rendesivir at this time.

**General and implementation considerations -** The inclusion of hospitalized patients in clinical studies of rendesivir is encouraged.

### Other treatments

**Recommendation 9.1 -** We recommend against the use convalescent plasma in patients hospitalized with COVID-19 (strong recommendation, moderate certainty of evidence).

**Recommendation 10.1 -** We suggest against the use ivermectin in patients hospitalized with COVID-19 (conditional recommendation, very low certainty of evidence).

**Recommendation 11.1 -** We recommend against the use colchicine in patients hospitalized with COVID-19 (strong recommendation, moderate certainty of evidence).

**Recommendation 12.1 -** We recommend against the use lopinavir/ritonavir in patients hospitalized with COVID-19 (strong recommendation, moderate certainty of evidence).

**Justification for the recommendation -** The panel of experts considered that, according to the available evidence, convalescent plasma, colchicine and lopinavir/ritonavir are not effective in the treatment of hospitalized patients with COVID-19 and are therefore not recommended.^(50-63)^ There are no studies that support the use of ivermectin in patients hospitalized with COVID-19, and its use should be restricted to clinical studies.

## DISCUSSION

In this guideline, which was developed by a panel of experts composed of representatives of medical societies and the Ministry of Health, 16 recommendations were elaborated, including treatment with corticosteroids for patients using supplemental oxygen and the use of anticoagulants at prophylactic doses for thromboembolism. In addition, the use of various drugs was discouraged.

During epidemics, when there are no clinical treatments with consolidated effectiveness, there is a tendency to use drugs based on the results of preclinical studies or observational studies with important limitations.^(13)^ Experience from epidemics has shown that these interventions may have a much lower benefit than expected, as was the case for oseltamivir during the swine flu epidemic in 2009. During the Ebola epidemic in 2014, several interventions were tested, including chloroquine, hydroxychloroquine, favipiravir immunobiologicals and convalescent plasma, none of which showed proof of effectiveness or safety.^(7)^

The understanding of SARS-CoV-2 infection and its treatment has evolved significantly over the past 12 months as a result of the collaborative efforts of several countries and research groups, which have developed randomized clinical studies evaluating potential candidates for the treatment of COVID-19.

Among them, the RECOVERY, SOLIDARITY, REMAP-CAP, and COALIZÃO studies in Brazil are noteworthy. As a result of these initiatives, some therapies with potential benefit, such as corticosteroids and tocilizumab,^(23,33)^ were identified, while several ineffective therapies, such as hydroxychloroquine, were discarded to promote safe and evidence-based treatment for the population and to promote the rational allocation of resources. Regarding costs, in terms of public health, it is important to note that in an epidemic scenario, the allocation of resources should prioritize interventions with greater certainty of benefit, such as the use of personal protective equipment, vaccines, interventions for the ventilatory support of patients and pharmacological therapies with proven effectiveness. The treatment of patients should be encouraged through research protocols with adequate design and potential to respond to society’s needs.

In addition to the evidence available in the scientific literature, the recommendations contained in the present guideline considered aspects relevant to the Brazilian reality, such as the availability of drugs in the national context (whether due to regulatory or accessibility factors), the acceptability of interventions to patients and health professionals and the costs associated with their use.

Thus, these recommendations are applicable to both the Unified Health System (SUS - *Sistema Único de Saúde*) and supplementary health services. Additionally, most of the recommendations in this document are aligned with therapeutic approaches recommended to date by major international organizations and societies, such as the WHO, NICE, NIH, IDSA and SSC.^(6,13,15,16)^

The present document consists of a joint positioning of seven medical societies, given the need to develop recommendations in a comprehensive manner and to contextualize them within different specialties in the face of the weaknesses of the available evidence and the relevance of the topic. With these recommendations, we hope to provide national guidance for the clinical practices related to pharmacological treatment for patients hospitalized with COVID-19, with the aim of promoting appropriate treatment and reducing the variability in the procedures applied.
