# Supplementary material for: Brazilian Guidelines for the pharmacological treatment of patients hospitalized with COVID-19 Joint guideline of Associação Brasileira de Medicina de Emergência, Associação de Medicina Intensiva Brasileira, Associação Médica Brasileira, Sociedade Brasileira de Angiologia e Cirurgia Vascular, Sociedade Brasileira de Infectologia, Sociedade Brasileira de Pneumologia e Tisiologia, Sociedade Brasileira de Reumatologia
Source: Rev Bras Ter Intensiva. 2022 Jan-Mar;34(1):1–12. doi: 10.5935/0103-507X.20220001-en (PMC9345589; doi:10.5935/0103-507X.20220001-en)
Supplement: Supplementary file 1 [file rbti-34-01-0001-suppl01.pdf]

Diretrizes Brasileiras para o tratamento farmacológico de pacientes hospitalizados com COVID-19. Diretriz conjunta da Associação Brasileira de Medicina de Emergência, Associação de Medicina Intensiva Brasileira, Associação Médica Brasileira, da Sociedade Brasileira de Angiologia e Cirurgia Vascular, da Sociedade Brasileira de Infectologia, da Sociedade Brasileira de Pneumologia e Tisiologia e da Sociedade Brasileira de Reumatologia

Brazilian Guidelines for the pharmacological treatment of patients hospitalized with COVID-19. *Joint guideline of Associação Brasileira de Medicina de Emergência, Associação de Medicina Intensiva Brasileira, Associação Médica Brasileira, Sociedade Brasileira de Angiologia e Cirurgia Vascular, Sociedade Brasileira de Infectologia, Sociedade Brasileira de Pneumologia e Tisiologia, Sociedade Brasileira de Reumatologia*

Maicon Falavigna[1,2], Cinara Stein[2], José Luiz Gomes do Amaral[3], Luciano Cesar Pontes de Azevedo[4,5], Karlyse Claudino Belli[2], Verônica Colpani[2], Clóvis Arns da Cunha[6,7], Felipe Dal-Pizzol[8], Maria Beatriz Souza Dias[5], Juliana Carvalho Ferreira[4,9,10], Ana Paula da Rocha Freitas[11,12], Débora Dalmas Gräf[2], Hélio Penna Guimarães[11], Suzana Margareth Ajeje Lobo[4,13], José Tadeu Monteiro[9], Michelle Silva Nunes[4,14], Maura Salaroli de Oliveira[5], Clementina Corah Lucas Prado[15], Vania Cristina Canuto Santos[15], Rosemeri Maurici da Silva[9], Marcone Lima Sobreira[16,17], Viviane Cordeiro Veiga[4,18], Ávila Teixeira Vidal[15], Ricardo Machado Xavier[19], Alexandre Prehn Zavascki[2], Flávia Ribeiro Machado[4,20], Carlos Roberto Ribeiro de Carvalho[5]

SUMÁRIO

Potenciais conflitos de interesses S2
Escopo do documento S3
Recomendações S5
Questão 1. Devemos utilizar corticosteroides em pacientes hospitalizados com COVID-19? S5
Questão 2. Devemos utilizar anticoagulantes em pacientes hospitalizados com COVID-19? S9
Questão 3. Devemos utilizar antimicrobianos em pacientes hospitalizados com COVID-19? S12
Questão 4. Devemos utilizar tocilizumabe em pacientes hospitalizados com COVID-19? S13
Questão 5. Devemos utilizar cloroquina ou hidroxicloroquina em pacientes hospitalizados com COVID-19? S15
Questão 6. Devemos utilizar azitromicina em pacientes hospitalizados com COVID-19? S16
Questão 7. Devemos utilizar casirivimabe + imdevimabe em pacientes hospitalizados com COVID-19? S18
Questão 8. Devemos utilizar rendesivir em pacientes hospitalizados com COVID-19? S18
Questão 9. Devemos utilizar plasma convalescente em pacientes hospitalizados com COVID-19? S22
Questão 10. Devemos utilizar ivermectina em pacientes hospitalizados com COVID-19? S22
Questão 11. Devemos utilizar colchicina em pacientes hospitalizados com COVID-19? S24
Questão 12. Devemos utilizar lopinavir/ritonavir em pacientes hospitalizados com COVID-19? S27
Referências S29

Potenciais conflitos de interesses

A lista a seguir (Tabela 1S) é referente aos potenciais conflitos de interesses do grupo de painelistas e metodologistas relacionados a esta diretriz. A avaliação dos possíveis conflitos de interesses foi determinada por um processo de revisão pelo grupo coordenador do documento.

Tabela 1S - Conflitos de interesse

Participante	Potencial conflito de interesse	Participação
Alexandre Zavascki	Recebe suporte financeiro para pesquisa do laboratório Pfizer sem relação com os medicamentos citados no artigo	Painelista
Ana Paula da Rocha Freitas	Não há conflitos de interesse	Painelista
Ávila Teixeira Vidal	Não há conflitos de interesse	Painelista
Carlos Roberto Ribeiro de Carvalho	Não há conflitos de interesse	Painelista
Clementina Corah Lucas Prado	Não há conflitos de interesse	Painelista
Clovis Arns da Cunha	Não há conflitos de interesse	Painelista
Cinara Stein*	Não há conflitos de interesse	Metodologista da diretriz
Débora Dalmas Gräf*	Não há conflitos de interesse	Metodologista da diretriz
Felipe Dal Pizzol	Não há conflitos de interesse	Painelista
Flávia Ribeiro Machado	Não há conflitos de interesse	Painelista
Hélio Penna Guimarães	Não há conflitos de interesse	Painelista
José Luis Gomes do Amaral	Não há conflitos de interesse	Painelista
José Tadeu Monteiro	Não há conflitos de interesse	Painelista
Juliana Carvalho Ferreira	Não há conflitos de interesse	Painelista
Karlyse Claudino Belli*	Não há conflitos de interesse	Metodologista da diretriz
Luciano Cesar Pontes Azevedo	Não há conflitos de interesse	Painelista
Maicon Falavigna*	Relacionados à diretriz: membro do comitê diretivo dos estudos COALIZÃO (não remunerado), avaliando hidroxicloroquina, azitromicina, corticosteroides e tocilizumabe, financiado pelo Ministério da Saúde, EMS e Hospitais Filantrópicos. Membro *GRADE Working Group*.<br>Não relacionados à diretriz: sócio da empresa de consultoria HTAnalyze, tendo executado e recebido honorários de projetos com Roche, PTC Therapeutics, Sanofi, Boehringer e Abbvie. Sócio da empresa de teleconsultoria em saúde Inova Médica	Metodologista da diretriz/ coordenador
Marcone Lima Sobreira	Não há conflitos de interesse	Painelista
Maria Beatriz Souza Dias	Não há conflitos de interesse	Painelista
Maura Salaroli de Oliveira	Não há conflitos de interesse	Painelista
Michelle Silva Nunes	Não há conflitos de interesse	Painelista
Ricardo Machado Xavier	Não relacionados à diretriz: palestrante/consultor dos laboratórios AbbVie, Pfizer e Novartis. Nenhum em relação aos medicamentos citados no artigo	Painelista
Suzana Lobo	Não há conflitos de interesse	Painelista
Rosemeri Maurici da Silva	Não há conflitos de interesse	Painelista
Vania Cristina Canuto Santos	Não há conflitos de interesse	Painelista
Viviane Cordeiro Veiga	Recebe suporte financeiro para pesquisa dos laboratórios Pfizer, Crisatália e Aspen	Painelista
Verônica Colpani*	Não há conflitos de interesse	Metodologista da diretriz

* Participantes não possuíam poder de voto.

Escopo do documento

Para identificar as questões clínicas de interesse, revisamos as tecnologias avaliadas em outras diretrizes nacionais e internacionais para o tratamento da COVID-19. A busca por diretrizes e recomendações foi realizada inicialmente por meio da plataforma e-COVID-19 RecMap, sendo avaliados os documentos originais sempre que necessário.[1,2] Foram selecionadas seis diretrizes contidas na plataforma, sendo acrescidas outras duas, por sugestão do grupo elaborador das recomendações.

As diretrizes que foram avaliadas para determinação de escopo estão apresentadas na tabela 2S.

Tabela 2S - Diretrizes consultadas para elaboração do documento

Organização	Título do documento	Data da versão acessada para elaboração da presente diretriz
World Health Organization	*Therapeutics and COVID-19 - Living Guideline*	Março de 2021
Australian National COVID-19 Clinical Evidence Taskforce	*Caring for People with COVID-19 - Supporting Australia's Healthcare Professionals with Continually Updated Evidence-Based Clinical Guidelines*	Abril de 2021
Infectious Diseases Society of America	*Infectious Diseases Society of America Guidelines on the Treatment and Management of Patients with COVID-19*	Abril de 2021
Associação de Medicina Intensiva Brasileira, Sociedade Brasileira de Infectologia, Sociedade Brasileira de Pneumologia e Tisiologia	Diretrizes para o tratamento farmacológico da COVID-19. Consenso da Associação de Medicina Intensiva Brasileira, da Sociedade Brasileira de Infectologia e da Sociedade Brasileira de Pneumologia e Tisiologia	Junho de 2020
National Institute for Health and Care Excellence	*COVID-19 Rapid Guideline: Managing COVID-19*	Março de 2021
Surviving Sepsis Campaign - Society of Critical Care Medicine/European Society of Intensive Care Medicine	*Surviving Sepsis Campaign Guidelines on the Management of Adults with Coronavirus Disease 2019 (COVID-19) in the ICU: First Update*	Março de 2021
National Institutes of Health	*COVID-19 Treatment Guidelines*	Abril de 2021
European Respiratory Society living guideline	*Management of Hospitalized Adults with Coronavirus Disease 2019 (COVID-19): a European Respiratory Society Living Guideline*	Abril de 2021

Fonte: elaboração própria.

Na tabela 3S, são apresentadas todas as tecnologias avaliadas por cada uma das diretrizes.

Tabela 3S - Tecnologias avaliadas pelas diretrizes consideradas para o presente estudo

Tecnologias	*Australian Taskforce*	*Infectious Diseases Society of America*	Diretrizes Brasileiras (Associação de Medicina Intensiva Brasileira, Sociedade Brasileira de Infectologia, Sociedade Brasileira de Pneumologia e Tisiologia)	*National Institute for Health and Care Excellence*	*Society of Critical Care Medicine*	*World Health Organization*	*National Institutes of Health*	*European Respiratory Society*	Total
Anakinra	✓						✓		2
Anticoagulantes	✓		✓		✓		✓	✓	5
Anticorpo monoclonal CT-P59	✓								1
Aprepitanto	✓								1
Azitromicina	✓		✓	✓				✓	4
Baloxavir marboxil	✓								1

Bamlanivimab	✓	✓					✓		3
Bamlanivimab + etesevimab									0
Baricitinib	✓								1
Baricitinib + rendesivir		✓					✓		2
Baricitinib + rendesivir + corticosteroides		✓							1
Budesonide	✓								1
Casirivimab + imdevimab							✓		1
Células-tronco mesenquimais de cordão umbilical humano	✓								1
Claritromicina	✓								1
Cloridrato de bromexina	✓								1
Cloroquina	✓	✓	✓		✓	✓	✓	✓	7
Cloroquina + azitromicina		✓	✓				✓	✓	4
Colchicina	✓			✓			✓	✓	4
Glicorticosteroides	✓	✓	✓	✓	✓	✓	✓	✓	8
Darunavir-cobicistat	✓								1
Doxiciclina	✓								1
Dutasteride	✓								1
Enisamium	✓								1
Esteroides (asma/DPOC)	✓								1
Fator estimulador de colônia de granulócitos humanos recombinante	✓								1
Favipiravir	✓								1
Fluvoxamine	✓						✓		2
Fomotidine		✓	✓	✓					3
IECA/BRA	✓								1
Imunoglobulina	✓						✓		2
IFN (B-1a, B-1b, gama, IFN-κ plus TFF2)	✓						✓	✓	3
Itraconazole	✓								1
Ivermectina	✓	✓				✓	✓		4
Levamisole	✓								1
Lopinavir/ritonavir (Kaletra®)	✓	✓	✓			✓	✓	✓	6
Metilprednisolona	✓								1
N-acetilcisteína	✓								1
Nitazoxanida	✓								1
Oseltamivir			✓						1
Peginterferon lambda	✓								1
Plasma	✓	✓			✓		✓		4
REGN-COV2	✓								1
Rendesivir	✓	✓		✓	✓	✓	✓	✓	7
Ruxolitinibe	✓						✓		2
Sarilumab	✓			✓			✓	✓	4
Sofosbuvir-daclatasvir 1	✓								1

Sulodexida	✓						1
Suplementação combinada de cofator metabólico	✓						1
Telmisartana	✓						1
Terapias contendo estrogênio	✓						1
Tocilizumabe	✓	✓	✓	✓	✓	✓	6
Tofacitinib					✓		1
Triazavirin	✓						1
Umifenovir	✓						1
Vitamin C	✓				✓		2
Vitamina D análagos (calcifediol/ colecalciferol)	✓			✓	✓		3
Zinco	✓				✓		2

DPOC - doença pulmonar obstrutiva crônica; IECA - inibidor da enzima conversora de angiotensina; BRA - bloqueador de receptor da angiotensina; IFN - interferon.
Fonte: elaboração própria.

Para o escopo do documento, o grupo selecionou por consenso as tecnologias a serem avaliadas com base em: frequência que eram apresentadas nas diferentes diretrizes; relevância para o contexto nacional. Assim, todas as tecnologias que foram avaliadas por pelo 50% das diretrizes foram consideradas, exceto o sarilumabe que não está disponível no Brasil. Adicionalmente, incluímos recomendações sobre casirivimabe + imdevimabe, devido à sua recente aprovação pela Agência Nacional de Vigilância Sanitária (Anvisa), e sobre o uso de anticoagulantes e antimicrobianos, devido à alta variabilidade na prática observada no contexto nacional.

Assim, as seguintes questões foram realizadas:

Questão 1 - Devemos utilizar corticosteroides em pacientes hospitalizados com COVID-19?
Questão 2 - Devemos utilizar anticoagulantes em pacientes hospitalizados com COVID-19?
Questão 3 - Devemos utilizar antimicrobianos em pacientes hospitalizados com covid-19?
Questão 4 - Devemos utilizar tocilizumabe em pacientes hospitalizados com COVID-19?
Questão 5 - Devemos utilizar cloroquina ou hidroxicloroquina em pacientes hospitalizados com COVID-19?
Questão 6 - Devemos utilizar azitromicina em pacientes hospitalizados com COVID-19?
Questão 7 - Devemos utilizar casirivimabe + imdevimabe em pacientes hospitalizados com COVID-19?
Questão 8 - Devemos utilizar rendesivir em pacientes hospitalizados com COVID-19?
Questão 9 - Devemos utilizar plasma convalescente em pacientes hospitalizados com COVID-19?
Questão 10 - Devemos utilizar ivermectina em pacientes hospitalizados com COVID-19?
Questão 11 - Devemos utilizar colchicina em pacientes hospitalizados com COVID-19?
Questão 12 - Devemos utilizar lopinavir/ritonavir em pacientes hospitalizados com COVID-19?

RECOMENDAÇÕES

Apresentamos as questões clínicas, as recomendações do painel de especialistas, as recomendações de outras diretrizes, um resumo das evidências e as tabelas de perfil de evidências, de acordo com a metodologia *Grading of Recommendations Assessment, Development and Evaluation* (GRADE).

Questão 1 - Devemos utilizar corticosteroides em pacientes hospitalizados com COVID-19?

Recomendação 1.1 - recomendamos o uso de dexametasona 6mg por via intravenosa (IV) ou via oral (VO), uma vez ao dia, por 10 dias, em pacientes hospitalizados com COVID-19 em uso de oxigênio suplementar (recomendação forte, certeza da evidência moderada).

Recomendação 1.2 - sugerimos não utilizar corticosteroides em pacientes hospitalizados com COVID-19 que não estão em uso de oxigênio suplementar (recomendação condicional, certeza da evidência baixa).

Recomendações de outras diretrizes

As diretrizes dividem as recomendações em grupos de pacientes em uso ou não de oxigênio suplementar, com algumas subdividindo os pacientes em uso de oxigênio suplementar de acordo com a gravidade (pacientes críticos, em geral em uso de ventilação mecânica - VM -, e pacientes graves, que necessitam de em uso de oxigênio suplementar para manter a saturação de oxigênio - SaO_2 adequada).

Das diretrizes consideradas, sete recomendaram o uso de corticosteroides, preferencialmente dexametasona, em pacientes com COVID-19 em suplementação de oxigênio, e não recomendaram o uso em pacientes que não estão em oxigenoterapia. Uma diretriz sugeriu não utilizar corticosteroides de rotina em pacientes com COVID-19, contudo sua recomendação foi realizada antes da publicação do estudo RECOVERY:

- *Australian Taskforce*: recomenda o uso de dexametasona 6mg uma vez ao dia IV ou VO, por até 10 dias (ou regime alternativo aceitável: hidrocortisona, prednisolona ou metilprednisolona), em pacientes adultos que estão fazendo uso de oxigênio suplementar, incluindo VM (recomendação forte, certeza da evidência moderada). Além disso, sugere não utilizar dexametasona de rotina em pacientes que não estão em suplementação de oxigênio (recomendação condicional, certeza da evidência baixa).

- *Infectious Diseases Society of America* (IDSA): recomenda o uso de dexametasona em pacientes em estágio crítico (na unidade de terapia intensiva – UTI – em uso de VM, oxigenação por membrana extracorporal – ECMO – ou em choque séptico; recomendação forte, certeza da evidência moderada). Sugere o uso de dexametasona em pacientes em estágio grave (SaO_2 < 94% em ar ambiente; recomendação condicional, certeza da evidência moderada) e sugere não usar dexametasona em pacientes não graves sem necessidade de suplementação de oxigênio (recomendação condicional, certeza da evidência baixa).

- Diretrizes Brasileiras (Associação de Medicina Intensiva Brasileira – AMIB -, Sociedade Brasileira de Infectologia - SBI, Sociedade Brasileira de Pneumologia e Tisiologia - SBPT): sugere não utilizar glicocorticosteroides de rotina no tratamento de pacientes com COVID-19 (recomendação condicional, certeza da evidência muito baixa). Recomendação realizada em maio de 2020, previamente à publicação do estudo RECOVERY, estando desatualizada.

- *National Institute for Health and Care Excellence* (NICE): recomenda o uso de dexametasona em pacientes que necessitam de oxigênio suplementar para atingir níveis adequados de SaO_2. Também recomenda o uso em pacientes com hipóxia que necessitam de oxigênio suplementar, mas que, por algum motivo, não podem receber essa terapia. Alternativamente, hidrocortisona ou prednisolona podem ser utilizadas quando a dexametasona não está disponível (recomendação forte, certeza da evidência moderada). Além disso, sugere não utilizar corticosteroides de rotina em pacientes que não necessitam de suplementação de oxigênio (recomendação condicional, certeza da evidência moderada).

- *Surviving Sepsis Campaign* (SSC): recomenda o uso de corticosteroides sistêmicos em pacientes adultos com COVID-19 grave ou crítica, por um período curto (recomendação forte, certeza da evidência moderada). Sugere o uso de dexametasona 6mg por 10 dias em pacientes adultos com COVID-19 grave ou crítica (recomendação condicional, certeza da evidência muito baixa).

- *World Health Organization* (WHO): recomenda o uso de corticosteroides sistêmicos em pacientes com COVID-19 grave ou crítica (recomendação forte, certeza da evidência moderada). Em pacientes com COVID-19 não grave, sugere não usar corticosteroides de rotina (recomendação condicional, certeza da evidência baixa).

- *National Institutes of Health* (NIH): recomenda o uso de dexametasona em pacientes hospitalizados com COVID-19 em suplementação de oxigênio por meio de cânula nasal (graduação BI), por meio de cânula nasal de alto fluxo (CNAF) ou ventilação não invasiva (VNI) (graduação AI) e em pacientes que necessitam de VM ou ECMO (graduação AI). Em caso de indisponibilidade da dexametasona, ela pode ser substituída por prednisona, metilprednisolona ou hidrocortisona (graduação BIII). Recomenda não utilizar dexametasona ou outros corticosteroides em pacientes com COVID-19 moderada sem suplementação de oxigênio (graduação AIIa);

- *European Respiratory Society* (ERS): recomenda o uso de corticosteroides em pacientes com COVID-19 em suplementação de oxigênio (recomendação forte, certeza da evidência moderada) e não recomenda o uso em pacientes hospitalizados sem suplementação de oxigênio (recomendação forte, certeza da evidência moderada).

Resumo das evidências

Na tomada de decisão, utilizamos as evidências apresentadas pela IDSA, datadas de 25 de setembro de 2020, que contempla as evidências de forma atualizada, não tendo sido identificadas novas evidências relevantes que pudessem ter impacto na tomada de decisão. Os perfis de evidências estão apresentados nas tabelas 4S a 6S, correspondendo a pacientes com doença crítica (uso de VM invasiva - VMI), com doença grave (em uso de oxigênio suplementar, mas não utilizando VM) e hospitalizados sem uso de oxigênio suplementar.

Tabela 4S - Perfil de evidências: devemos utilizar corticosteroides em pacientes críticos hospitalizados com COVID-19?

Avaliação da certeza							Sumário de resultados				
Participantes nos estudos (n) Seguimento	Risco de viés	Inconsistência	Evidência indireta	Imprecisão	Viés de publicação	Qualidade geral da evidência	Taxas de eventos do estudo (%)		Efeito relativo (IC95%)	Efeitos absolutos potenciais	
							Com controle	Com corticosteroides		Risco com controle	Diferença de risco com corticosteroides
Mortalidade (seguimento: 28 dias)											
1.844 (7 ECRs)	Não grave	Não grave	Não grave	Não grave	Nenhum	⊕⊕⊕⊕ Alta	485/1.095 (44,3%)	280/749 (37,4%)	RC = 0,66 (0,54 - 0,82)	443 por 1.000	99 menos por 1.000 (de 143 menos para 48 menos)
Alta hospitalar (seguimento: 28 dias)											
6.425 (1 ECR)	Não grave*	Não grave	Grave†	Não grave	Nenhum	⊕⊕⊕◯ Moderada	2.639/4321 (61,1%)	1.360/2.104 (64,6%)	RR = 1,11 (1,04 - 1,19)	611 por 1.000	67 mais por 1.000 (de 24 mais para 116 mais)
Eventos adversos graves											
696 (6 ECRs)	Não grave	Não grave	Não grave	Grave‡	Nenhum	⊕⊕⊕◯ Moderada	Seis estudos reportaram 64 eventos entre 354 pacientes randomizados para o Grupo Corticosteroides e 80 eventos entre 342 pacientes randomizados para o Grupo Controle (Stem 2020)				

IC95% - intervalo de confiança de 95%; ECR - ensaio clínico randomizado; RC - razão de chances; RR – risco relativo. * Análise ajustada para idade basal; † evidência indireta devido a diferentes sistemas de saúde (alocação de recursos de terapia intensiva em um estudo não cego). Evidência indireta para outros corticosteroides; ‡ intervalo de confiança inclui potencial risco e benefício. Ainda, poucos eventos relatados não atendem ao tamanho ótimo da informação e sugerem fragilidade na estimativa.

Referências

1. The WHO Rapid Evidence Appraisal for COVID-19 Therapies Working Group, Sterne JA, Murthy S, Diaz JV, Slutsky AS, Villar J, Angus DC, et al. Association Between Administration of Systemic Corticosteroids and Mortality Among Critically Ill Patients With COVID-19: A Meta-analysis. JAMA. 2020;324(13):1330-41.[3]
2. RECOVERY Collaborative Group, Horby P, Lim WS, Emberson J, Mafham M, Bell JL, Linsell L, et al. Dexamethasone in hospitalized patients with COVID-19. N Engl J Med. 2021;384(8):693-704.[4]

Fonte: adaptada de Bhimraj A, Morgan RL, Shumaker AH, Lavergne V, Baden L, Cheng VC, Edwards KM, Gandhi R, Gallagher J, Muller WJ, O'Horo JC, Shoham S, Murad MH, Mustafa RA, Sultan S, Falck-Ytter Y. Infectious Diseases Society of America. Guidelines on the Treatment and Management of Patients with COVID-19: Infectious Diseases Society of America; 2021; Version 5.6.0. [cited 2021 May 10]. Available from: https://www.idsociety.org/practice-guideline/covid-19-guideline-treatment-and-management/ [5]

Em pacientes críticos (em uso de VM), sete estudos foram identificados, mostrando importante redução na mortalidade (razão de chance - RC - de 0,66; intervalo de confiança de 95% - IC95% - 0,54 - 0,82). A maioria dos pacientes avaliados foi procedente do estudo RECOVERY, o qual avaliou 1.007 pacientes em VM, randomizados para o uso de dexametasona na dose de 6mg ao dia, por 10 dias, ou para o grupo controle, sendo observada redução relativa de risco de 36% com o uso de corticosteroides (mortalidade de 29,3% *versus* 41,4%; risco relativo - RR - de 0,64; IC95% 0,51 - 0,81).[3] Demais estudos clínicos seguem resultados semelhantes.[3]

O RECOVERY também identificou redução de mortalidade em pacientes em uso de oxigênio suplementar, que não estavam em uso VM (mortalidade de 23,3% versus 26,2%; RR 0,82; IC95% 0,72 - 0,94). Contudo, em pacientes hospitalizados sem necessidade de oxigênio suplementar, os pacientes em uso de corticosteroides tiveram piores resultados do que aqueles do grupo controle (mortalidade de 17,8% versus 14,0%; RR 1,19; IC95% 0,92 - 1,19).[3]

Tabela 5S - Perfil de evidências: devemos utilizar corticosteroides em pacientes graves hospitalizados com COVID-19?

Avaliação da certeza							Sumário de resultados				
							Taxas de eventos do estudo (%)			Efeitos absolutos potenciais	
**Participantes nos estudos (n) Seguimento**	**Risco de viés**	**Inconsistência**	**Evidência indireta**	**Imprecisão**	**Viés de publicação**	**Avaliação da certeza**	**Com controle**	**Com corticosteroides**	**Efeito relativo (IC95%)**	**Risco com controle**	**Diferença de risco com corticosteroides**
Mortalidade (seguimento: 28 dias)											
6.425 (1 ECR)	Não grave*	Não grave	Grave†	Não grave	Nenhum	⨁⨁⨁◯ Moderada	1.065/4.321 (24,6%)	454/2.104 (21,6%)	RR = 0,83 (0,74 - 0,92)	246 por 1.000	42 menos por 1.000 (de 64 menos para 20 menos)
Alta hospitalar (seguimento: 28 dias)											
6.425 (1 ECR)	Não grave*	Não grave	Grave†	Não grave	Nenhum	⨁⨁⨁◯ Moderada	2.639/4.321 (61,1%)	1.360/2.104 (64,6%)	RR = 1,11 (1,04 - 1,19)	611 por 1.000	67 mais por 1.000 (de 24 mais para 116 mais)
Eventos adversos											
							Pacientes que receberam corticosteroides por um curto período apresentaram hiperglicemia, eventos adversos neurológicos (por exemplo: agitação/confusão), supressão adrenal e risco de infecção (Salton 2020; Henzen 2000; Siemieniuk 2015)				

IC95% - intervalo de confiança de 95%; ECR - ensaio clínico randomizado; RR - risco relativo. * Análise ajustada para idade basal; † evidência indireta devido a diferentes sistemas de saúde (alocação de recursos de terapia intensiva em um estudo não cego). Evidência indireta para outros corticosteroides.

Referência
1. RECOVERY Collaborative Group, Horby P, Lim WS, Emberson J, Mafham M, Bell JL, Linsell L, et al. Dexamethasone in hospitalized patients with COVID-19. N Engl J Med. 2021;384(8):693-704.[4]
Fonte: adaptada de Bhimraj A, Morgan RL, Shumaker AH, Lavergne V, Baden L, Cheng VC, Edwards KM, Gandhi R, Gallagher J, Muller WJ, O'Horo JC, Shoham S, Murad MH, Mustafa RA, Sultan S, Falck-Ytter Y. Infectious Diseases Society of America. Guidelines on the Treatment and Management of Patients with COVID-19: Infectious Diseases Society of America; 2021; Version 5.6.0. [cited 2021 May 10]. Available from: https://www.idsociety.org/practice-guideline/covid-19-guideline-treatment-and-management/ [5]

Tabela 6S - Perfil de evidências: devemos utilizar corticosteroides em pacientes hospitalizados com COVID-19 não recebendo oxigênio suplementar?

Avaliação da certeza							Sumário de resultados				
							Taxas de eventos do estudo (%)			Efeitos absolutos potenciais	
**Participantes nos estudos (n) Seguimento**	**Risco de viés**	**Inconsistência**	**Evidência indireta**	**Imprecisão**	**Viés de publicação**	**Qualidade geral da evidência**	**Com controle**	**Com corticosteroides**	**Efeito relativo (IC95%)**	**Risco com controle**	**Diferença de risco com corticosteroides**
Mortalidade (seguimento: 28 dias)											
1.535 (1 ECR)	Grave*	Não grave	Não grave	Grave†	Nenhum	⨁⨁◯◯ Baixa	137/1.034 (13,2%)	85/501 (17,0%)	RR = 1,22 (0,93 - 1,61)	132 por 1.000	29 mais por 1.000 (de 9 menos para 81 mais)
Alta hospitalar (seguimento: 28 dias)											
1.535 (1 ECR)	Grave*	Não grave	Não grave	Grave‡	Nenhum	⨁⨁◯◯ Baixa	791/1.034 (76,5%)	366/501 (73,1%)	RR = 0,99 (0,87 - 1,12)	765 por 1.000	8 menos por 1.000 (de 99 menos para 92 mais)
Eventos adversos											
							Pacientes que receberam corticosteroides por um curto período apresentaram hiperglicemia, eventos adversos neurológicos (por exemplo: agitação/confusão), supressão adrenal e risco de infecção (Salton 2020; Henzen 2000; Siemieniuk 2015)				

ECR - ensaio clínico randomizado; IC95% - intervalo de confiança de 95%; RR - risco relativo. * Risco de viés devido a efeito de subgrupo entre os pacientes que não receberam oxigênio suplementar; † evidência indireta devido a diferentes sistemas de saúde (alocação de recursos de terapia intensiva em um estudo não cego). Evidência indireta para outros corticosteroides; ‡ o intervalo de confiança de 95% não exclui potencial risco e benefício.

Referência
1.RECOVERY Collaborative Group, Horby P, Lim WS, Emberson J, Mafham M, Bell JL, Linsell L, et al. Dexamethasone in hospitalized patients with COVID-19. N Engl J Med. 2021;384(8):693-704.[4]
Fonte: adaptada de Bhimraj A, Morgan RL, Shumaker AH, Lavergne V, Baden L, Cheng VC, Edwards KM, Gandhi R, Gallagher J, Muller WJ, O'Horo JC, Shoham S, Murad MH, Mustafa RA, Sultan S, Falck-Ytter Y. Infectious Diseases Society of America. Guidelines on the Treatment and Management of Patients with COVID-19: Infectious Diseases Society of America; 2021; Version 5.6.0. [cited 2021 May 10]. Available from: https://www.idsociety.org/practice-guideline/covid-19-guideline-treatment-and-management/[5]

Questão 2 - Devemos utilizar anticoagulantes em pacientes hospitalizados com COVID-19?

Recomendação 2.1 - recomendamos o uso de anticoagulantes em doses de profilaxia para tromboembolismo venoso (TEV) em pacientes críticos (em uso de drogas vasoativas, terapia de substituição renal, CNAF, VNI ou VMI) com COVID-19 (recomendação não graduada).

Recomendação 2.2 - sugerimos não utilizar doses intermediárias ou anticoagulação terapêutica em pacientes críticos (em uso de drogas vasoativas, terapia de substituição renal, CNAF, VNI ou VMI) com COVID-19 sem evidência de tromboembolismo (recomendação condicional, certeza da evidência muito baixa).

Recomendação 2.3 - sugerimos utilizar heparina ou enoxaparina em doses terapêuticas em pacientes não críticos (sem necessidade de drogas vasoativas, terapia de substituição renal, CNAF, VNI ou VMI), hospitalizados com COVID-19 (recomendação condicional, certeza da evidência muito baixa).

Recomendações de outras diretrizes

Seis diretrizes sugerem ou recomendam o uso de doses profiláticas de anticoagulantes em pacientes com COVID-19 e não recomendam o uso terapêutico rotineiro desses medicamentos:

- *American Society of Hematology* (ASH): sugere o uso de anticoagulação de intensidade profilática em vez de intensidade intermediária ou terapêutica em pacientes com doença crítica relacionada à COVID-19 que não possuem suspeita ou confirmação de tromboembolismo venoso (TEV) (recomendação condicional, certeza da evidência muito baixa).

- *Australian Taskforce*: sugere o uso de doses profiláticas de anticoagulantes, de preferência heparina de baixo peso molecular (por exemplo: enoxaparina 40mg uma vez ao dia, ou dalteparina 5.000UI uma vez ao dia) em adultos com COVID-19 moderada, grave ou crítica ou outras indicações, a menos que haja uma contraindicação, tal como risco de sangramento importante. Quando a taxa de filtração glomerular estimada (eTFG) for inferior a 30mL/minuto/1,73m^2, heparina não fracionada ou doses ajustadas de depuração de heparina de baixo peso molecular podem ser usadas (por exemplo: enoxaparina 20mg uma vez ao dia ou dalteparina 2.500 UI uma vez ao dia; consenso de especialistas). Sugere não usar rotineiramente doses terapêuticas de anticoagulantes em pacientes adultos com COVID-19 grave ou crítica. Não há indicação adicional para dosagem terapêutica de anticoagulantes em adultos com COVID-19 grave ou crítica além das melhores práticas atuais (recomendação condicional, certeza da evidência muito baixa).

- Diretrizes brasileiras (AMIB, SBI e SBPT): recomendam o uso rotineiro de doses profiláticas de anticoagulantes em pacientes hospitalizados com COVID-19 (recomendação forte; certeza da evidência muito baixa). Sugere o uso rotineiro de doses terapêuticas de heparina para o tratamento de COVID-19 (recomendação condicional; certeza da evidência muito baixa).

- SSC: recomenda o uso de tratamento farmacológico para profilaxia de TEV em pacientes graves ou críticos com COVID-19 (recomendação forte, certeza da evidência moderada). Sugere não usar rotineiramente anticoagulantes de forma terapêutica fora do ambiente de ensaios clínicos em pacientes graves ou críticos com COVID-19 e sem evidência de TEV (recomendação condicional, certeza da evidência muito baixa).

- NIH: pacientes hospitalizados com COVID-19 não devem receber alta hospitalar rotineiramente durante a profilaxia de TEV (graduação AIII). A continuação da anticoagulação com um regime aprovado pela *Food and Drug Administration* para profilaxia estendida de TEV após a alta hospitalar pode ser considerada para pacientes com baixo risco de sangramento e alto risco de TEV, de acordo com os protocolos para pacientes sem COVID-19 (graduação BI). Adultos não gestantes hospitalizados com COVID-19 devem receber anticoagulação em dose profilática (graduação AIII).

- ERS: recomenda oferecer uma forma de anticoagulação para pacientes hospitalizados com COVID-19 (recomendação forte, certeza da evidência muito baixa), contudo o painel não foi capaz de realizar recomendação em relação ao medicamento a ser utilizado ou à dose (profilaxia, intermediária ou anticoagulação terapêutica).

Resumo das evidências

Na tomada de decisão, utilizamos as evidências apresentadas pelo *Australian Taskforce*, datadas de 14 de outubro de 2021, que contemplam as evidências de forma atualizada. Adicionalmente, foi incluída uma publicação identificada pelos especialistas.[7] O perfil de evidências é apresentado nas tabelas 7S e 8S, correspondendo a pacientes críticos que fizeram uso de heparina ou enoxaparina e não críticos com COVID-19 que fizeram uso de heparina, respectivamente.

Tabela 7S - **Perfil de evidências: devemos utilizar anticoagulantes (heparina ou enxoparina) em pacientes críticos com COVID-19?**

Certeza da evidência							Sumário de resultados				
							Taxas de eventos do estudo (%)			Efeitos absolutos potenciais	
Participantes nos estudos (n) Seguimento	Risco de viés	Inconsistência	Evidência indireta	Imprecisão	Viés de publicação	Avaliação da certeza da evidência	Com tromboprofilaxia em dose profilática	Com tromboprofilaxia em dose terapêutica	Efeito relativo (IC95%)	Risco com tromboprofilaxia em dose profilática	Diferença de risco com tromboprofilaxia em dose terapêutica
Mortalidade por todas as causas (seguimento: média 28 - 30 dias)											
1.853 (4 ECRs)	Grave*	Não grave	Não grave	Não grave	Nenhum	⊕⊕⊕◯ Moderada	338/946 (35,7%)	332/907 (36,6%)	RR = 1,03 (0,91 - 1,17)	357 por 1.000	10 mais por 1.000 (de 31 menos para 58 mais)
Eventos trombóticos											
1.824 (3 ECRs)	Grave*	Não grave	Não grave	Grave†	Nenhum	⊕⊕◯◯ Baixa	55/931 (5,9%)	77/893 (8,6%)	RR = 0,81 (0,51 - 1,28)	59 por 1.000	15 menos por 1.000 (de 38 menos para 22 mais)
Sangramento maior											
1.929 (5 ECRs)	Grave*	Não grave	Não grave	Grave†	Nenhum	⊕⊕◯◯ Baixa	19/982 (1,9%)	33/947 (3,5%)	RR = 1,70 (0,97 - 2,98)	19 por 1.000	14 mais por 1.000 (de 1 menos para 38 mais)
Desfecho composto (tromboembolismo arterial, tromboembolismo venoso, ou morte)											
83 (1 ECR)	Não grave	Não grave	Não grave	Muito grave†‡	Nenhum	⊕⊕◯◯ Baixa	21/38 (55,3%)	23/45 (51,1%)	RR = 0,92 (0,62 - 1,39)	553 por 1.000	44 menos por 1.000 (de 210 menos para 216 mais)

IC95% - intervalo de confiança de 95%; ECR - ensaio clínico randomizado; RR - risco relativo. * Inadequada alocação sigilosa durante o processo de randomização; † intervalo de confiança amplo; ‡ dados de um único estudo.

Referências

1. Lemos AC, do Espírito Santo DA, Salvetti MC, Gilio RN, Agra LB, Pazin-Filho A, et al. Therapeutic versus prophylactic anticoagulation for severe COVID-19: a randomized phase II clinical trial (HESACOVID). Thromb Res. 2020;196:359-66.[6]
2. INSPIRATION Investigators, Sadeghipour P, Talasaz AH, Rashidi F, Sharif-Kashani B, Beigmohammadi MT, Farrokhpour M, et al. Effect of intermediate-dose vs standard-dose prophylactic anticoagulation on thrombotic events, extracorporeal membrane oxygenation treatment, or mortality among patients with COVID-19 admitted to the intensive care unit: the INSPIRATION randomized clinical trial. JAMA. 2021;325(16):1620-30.[7]
3. Perepu US, Chambers I, Wahab A, Ten Eyck P, Wu C, Dayal S, et al. Standard prophylactic versus intermediate dose enoxaparin in adults with severe COVID-19: a multi-center, open-label, randomized controlled trial. J Thromb Haemost. 2021;19(9):2225-34.[8]
4. REMAP-CAP Investigators; ACTIV-4a Investigators; ATTACC Investigators, Goligher EC, Bradbury CA, McVerry BJ, et al. Therapeutic anticoagulation with heparin in critically ill patients with Covid-19. N Engl J Med. 2021;385(9):777-89.[9]
5. Spyropoulos AC, Goldin M, Giannis D, Diab W, Wang J, Khanijo S, Mignatti A, Gianos E, Cohen M, Sharifova G, Lund JM, Tafur A, Lewis PA, Cohoon KP, Rahman H, Sison CP, Lesser ML, Ochani K, Agrawal N, Hsia J, Anderson VE, Bonaca M, Halperin JL, Weitz JI; HEP-COVID Investigators. Efficacy and safety of therapeutic-dose heparin vs standard prophylactic or intermediate-dose heparins for thromboprophylaxis in high-risk hospitalized patients with COVID-19: the HEP-COVID randomized clinical trial. JAMA Intern Med. 2021 Oct 7:e216203.[10]

Fonte: adaptada de National COVID-19 Clinical Evidence Taskforce. Caring for people with COVID-19. Supporting Australia's healthcare professionals with continually updated evidence-based clinical guidelines. 2021. [cited 2021 May10]. Available from: https://covid19evidence.net.au/#living-guidelines.[11]

Tabela 8S - Perfil de evidências: devemos utilizar heparina em pacientes não críticos com COVID-19?

Certeza da evidência							Sumário de resultados				
							Taxas de eventos do estudo (%)			Efeitos absolutos potenciais	
Participantes nos estudos (n) Seguimento	Risco de viés	Inconsistência	Evidência indireta	Imprecisão	Viés de publicação	Avaliação da certeza da evidência	Com heparina em dose profilática	Com heparina em dose terapêutica	Efeito relativo (IC95%)	Risco com heparina em dose profilática	Diferença de risco com heparina em dose terapêutica
Mortalidade por todas as causas											
2.691 (2 ECRs)	Grave*	Grave†	Não grave	Grave‡	Nenhum	⊕◯◯◯ Muito baixa	104/1.283 (8,1%)	90/1.408 (6,4%)	RR = 0,50 (0,13 - 1,86)	81 por 1.000	41 menos por 1.000 (de 71 menos para 70 mais)
Eventos trombóticos											
2.691 (2 ECRs)	Grave*	Não grave	Não grave	Não grave	Nenhum	⊕⊕⊕◯ Moderada	29/1.283 (2,3%)	15/1.408 (1,1%)	RR = 0,48 (0,26 - 0,89)	23 por 1.000	12 menos por 1.000 (de 17 menos para 2 menos)
Hemorragia grave											
2.862 (3 ECRs)	Grave*	Não grave	Não grave	Grave‡	Nenhum	⊕⊕◯◯ Baixa	15/1.370 (1,1%)	26/1.492 (1,7%)	RR = 1,43 (0,62 - 3,29)	11 por 1.000	5 mais por 1.000 (de 4 menos para 25 mais)
Sem necessidade de suplementação de oxigênio (ventilação mecânica invasiva ou não invasiva, oxigênio nasal de alto fluxo, terapia vasopressora ou suporte de ECMO; seguimento: variação 21 dias para 28 dias)											
2.692 (2 ECRs)	Não grave	Não grave	Não grave	Grave§	Nenhum	⊕⊕⊕◯ Moderada	ATTACC, ACTIV-4a e REMAP-CAP 2021: anticoagulação terapêutica foi superior ao tratamento profilático em 98,6% (razão de chance ajustada de 1,27; intervalo de credibilidade de 95%, 1,03-1,58) RAPID 2021: observou que o número médio de dias livres foi de 25,8 (DP de 6,2) no grupo de heparina terapêutica e 24,1 (DP de 8,8) no grupo de heparina profilática (razão de chance de 1,41; IC95% 0,90 - 2,21; p = 0,13) após 28 dias de acompanhamento				
Desfecho composto (tromboembolismo venoso, tromboembolismo arterial, ou morte)											
170 (1 ECR)	Não grave	Não grave	Não grave	Grave¶	Nenhum	⊕⊕⊕◯ Moderada	31/86 (36,0%)	14/84 (16,7%)	RR = 0,46 (0,27 - 0,81)	360 por 1.000	195 menos por 1.000 (de 263 menos para 68 menos)

IC95% - intervalo de confiança de 95%; ECR - ensaio clínico randomizado; RR - risco relativo; DP - desvio-parão. * Inadequada alocação sigilosa durante o processo de randomização; † as estimativas pontuais variam amplamente. A magnitude da heterogeneidade estatística foi alta, com I2: 83%. O intervalo de confiança não se sobrepõe, e a direção do efeito não é consistente entre os estudos incluídos; ‡ intervalo de confiança amplo; § a direção do efeito não é consistente entre os estudos incluídos; ¶ dados de um único estudo.

Referências

1. Sholzberg M, Tang GH, Rahhal H, AlHamzah M, Kreuziger LB, Áinle FN, Alomran F, Alayed K, Alsheef M, AlSumait F, Pompilio CE, Sperlich C, Tangri S, Tang T, Jaksa P, Suryanarayan D, Almarshoodi M, Castellucci LA, James PD, Lillicrap D, Carrier M, Beckett A, Colovos C, Jayakar J, Arsenault MP, Wu C, Doyon K, Andreou ER, Dounaevskaia V, Tseng EK, Lim G, Fralick M, Middeldorp S, Lee AYY, Zuo F, da Costa BR, Thorpe KE, Negri EM, Cushman M, Jüni P; RAPID trial investigators. Effectiveness of therapeutic heparin versus prophylactic heparin on death, mechanical ventilation, or intensive care unit admission in moderately ill patients with covid-19 admitted to hospital: RAPID randomised clinical trial. BMJ. 2021;375:n2400.[12]
2. Lopes RD, de Barros e Silva PG, Furtado RH, Macedo AV, Bronhara B, Damiani LP, Barbosa LM, de Aveiro Morata J, Ramacciotti E, de Aquino Martins P, de Oliveira AL, Nunes VS, Ritt LE, Rocha AT, Tramujas L, Santos SV, Diaz DR, Viana LS, Melro LM, de Alcântara Chaud MS, Figueiredo EL, Neuenschwander FC, Dracoulakis MD, Lima RG, de Souza Dantas VC, Fernandes AC, Gebara OC, Hernandes ME, Queiroz DA, Veiga VC, Canesin MF, de Faria LM, Feitosa-Filho GS, Gazzana MB, Liporace IL, de Oliveira Twardowsky A, Maia LN, Machado FR, de Matos Soeiro A, Conceição-Souza GE, Armaganijan L, Guimarães PO, Rosa RG, Azevedo LC, Alexander JH, Avezum A, Cavalcanti AB, Berwanger O; ACTION Coalition COVID-19 Brazil IV Investigators. Therapeutic versus prophylactic anticoagulation for patients admitted to hospital with COVID-19 and elevated D-dimer concentration (ACTION): an open-label, multicentre, randomised, controlled trial. Lancet. 2021;397(10291):2253-63.[13]
3. REMAP-CAP Investigators; ACTIV-4a Investigators; ATTACC Investigators, Goligher EC, Bradbury CA, McVerry BJ, et al. Therapeutic anticoagulation with heparin in critically ill patients with Covid-19. N Engl J Med. 2021;385(9):777-89.[9]
4. Spyropoulos AC, Goldin M, Giannis D, Diab W, Wang J, Khanijo S, Mignatti A, Gianos E, Cohen M, Sharifova G, Lund JM, Tafur A, Lewis PA, Cohoon KP, Rahman H, Sison CP, Lesser ML, Ochani K, Agrawal N, Hsia J, Anderson VE, Bonaca M, Halperin JL, Weitz JI; HEP-COVID Investigators. Efficacy and safety of therapeutic-dose heparin vs standard prophylactic or intermediate-dose heparins for thromboprophylaxis in high-risk hospitalized patients with COVID-19: the HEP-COVID randomized clinical trial. JAMA Intern Med. 2021 Oct 7:e216203.[10]

Fonte: adaptada de National COVID-19 Clinical Evidence Taskforce. Caring for people with COVID-19. Supporting Australia's healthcare professionals with continually updated, evidence-based clinical guidelines. 2021. [cited 2021 May10]. Available from: https://covid19evidence.net.au/#living-guidelines.[11]

Cinco ensaios clínicos randomizados[6-10] avaliaram o uso de anticoagulantes em pacientes críticos com COVID-19. Não houve diferença na mortalidade por todas as causas (RR de 1,03; IC95% 0,91 - 1,17),[6-9] eventos trombóticos (RR de 0,81; IC95% 0,51 - 1,28),[7-9] sangramento maior (RR de 1,70; IC95% 0,97 - 2,98)[6-10] e desfecho composto, que corresponde tromboembolismo arterial, TEV ou morte (RR de 0,92; IC95% 0,62 - 1,39),[7] em pacientes utilizando anticoagulação terapêutica em comparação a profilaxia padrão.

Em relação ao uso de anticoagulantes em pacientes não críticos com COVID-19, foram encontrados quatro ensaios clínicos randomizados.[10,12-14] Em análise de subgrupo por tipo de medicamento, quando utilizada rivaroxabana, não houve diferença na mortalidade por todas as causas (RR de 1,49; IC95% 0,90 - 2,46),[13] eventos trombóticos (RR de 0,75; IC95% 0,45 - 1,26)[13] e sangramento maior (RR de 2,45; IC95% 0,78 - 7,73),[13] quando comparada à anticoagulação terapêutica em relação à profilaxia padrão. Quando utilizada heparina, não houve diferença na mortalidade por todas as causas (RR de 0,50; IC95% 0,13 - 1,86)[12,14] e por sangramento maior (RR de 1,43; IC 0,62 - 3,29),[10,12,14] porém houve redução do risco de eventos trombóticos em 52% (RR de 0,48; IC95% 0,26 - 0,89)[12,14] e eventos para o desfecho composto que corresponde a tromboembolismo arterial, TEV ou morte (RR de 0,46; IC95% 0,27 - 0,81),[7] quando comparada anticoagulação terapêutica em relação à profilaxia padrão. Em relação ao desfecho dias livres de suporte orgânico, na análise conjunta dos estudos ATTACC, ACTIV-4a, e REMAP-CAP,[12] a anticoagulação terapêutica foi superior ao tratamento profilático em 98,6% (RC ajustada de 1,27; intervalo de credibilidade de 95%, 1,03 - 1,58). Já o estudo RAPID[14] observou que o número médio de dias livres foi de 25,8 (desvio-padrão - DP - de 6,2) no grupo de heparina terapêutica e 24,1 (DP de 8,8) no grupo de heparina profilática (RC de 1,41; IC95% 0,90 - 2,21; p = 0,13) após 28 dias de acompanhamento.

Dois estudos[7,9] contribuíram com maior peso na análise quando avaliado o uso de anticoagulantes em pacientes críticos com COVID-19. Na análise conjunta dos estudos REMAP-CAP, ACTIV-4a e ATTACC,[9] foram randomizados 1.207 pacientes críticos com COVID-19 recebendo suporte ventilatório por CNAF, VNI ou VM, randomizados para anticoagulação terapêutica ou para profilaxia padrão. O estudo INSPIRATION[7] avaliou os efeitos da dose intermediária (enoxaparina, 1mg/kg diário) em comparação à profilática (enoxaparina, 40mg/dia) de anticoagulação, em 600 pacientes críticos com COVID-19 admitidos em UTI.

Já em relação aos estudos que avaliaram o uso de anticoagulantes em pacientes não críticos com COVID-19, dois estudos[13,14] contribuíram com maior peso na análise. Na análise dos estudos ATTACC, ACTIV-4a e REMAP-CAP,[14] foram randomizados 2.244 pacientes não críticos com COVID-19 que não faziam uso de CNAF, VNI ou VM para receber regimes pragmaticamente definidos de anticoagulação de dose terapêutica com heparina ou tromboprofilaxia farmacológica de tratamento padrão. O estudo ACTION[13] avaliou os efeitos de doses terapêuticas (pacientes estáveis: rivaroxabana oral 20mg ou 15mg diária; pacientes instáveis: dose inicial de enoxaparina subcutânea 1mg/kg duas vezes ao dia, ou heparina não fracionada intravenosa; ambas seguidas de até 30 dias) em comparação a profiláticas (enoxaparina ou heparina não fracionada) de anticoagulantes em 615 pacientes não críticos com COVID-19.[15]

Questão 3 - Devemos utilizar antimicrobianos em pacientes hospitalizados com COVID-19?

Recomendação 3.1 - recomendamos não utilizar antimicrobianos em pacientes com COVID-19 sem suspeita de infecção bacteriana (recomendação não graduada).

Recomendações de outras diretrizes

Das diretrizes consideradas, apenas duas avaliaram o uso de antimicrobianos em pacientes com COVID-19:

- Diretrizes brasileiras (AMIB, SBI e SBPT): recomendam o uso de antibacterianos em pacientes com COVID-19 com suspeita da infecção bacteriana (recomendação não graduada).

- NICE: não indica o uso de antibióticos para prevenir ou tratar pacientes com COVID-19 (recomendação não graduada).

Resumo das evidências

Não foram encontradas evidências sobre o uso de antimicrobianos em pacientes hospitalizados. A taxa de coinfecção e de infecção secundária em pacientes com COVID-19 é relativamente baixa.[17]

Questão 4 - Devemos utilizar tocilizumabe em pacientes hospitalizados com COVID-19?

Recomendação 4.1 - é clinicamente indicada a utilização do tocilizumabe em pacientes hospitalizados com COVID-19 em uso de VNI ou CNAF, contudo não é possível recomendá-lo no momento (maio de 2021), pois não há aprovação em bula para essa indicação, e há incertezas no acesso ao medicamento, devido à indisponibilidade para suprir a demanda potencial (sem recomendação, certeza da evidência moderada).

Recomendação 4.2 - sugerimos não utilizar tocilizumabe em pacientes em VM (recomendação condicional, certeza da evidência moderada).

Recomendações de outras diretrizes

Das diretrizes consideradas, seis avaliaram o tocilizumabe, sendo que duas recomendam o uso de tocilizumabe em pacientes hospitalizados com COVID-19, três sugerem o uso de tocilizumabe em pacientes hospitalizados com COVID-19 e uma sugere não utilizar tocilizumabe de rotina no tratamento da COVID-19. As diretrizes que indicam o uso possuem recomendações mais recentes, contemplando estudos recentemente publicados:

***- Australian Taskforce*:** sugere o uso de tocilizumabe para o tratamento de pacientes com COVID-19 que requerem oxigênio suplementar, particularmente se houver evidência de inflamação sistêmica (recomendação condicional, certeza de evidência moderada).

- IDSA: sugere o uso de tocilizumabe adicionado ao tratamento padrão (ou seja, esteroides) em pacientes hospitalizados com COVID-19 grave ou crítico, que apresentam marcadores elevados de inflamação sistêmica (recomendação condicional, certeza de evidência baixa).

- Diretrizes brasileiras (AMIB, SBI e SBPT): sugerem não utilizar tocilizumabe de rotina no tratamento da COVID-19 (recomendação fraca, certeza de evidência muito baixa).

- NICE: recomenda o uso de tocilizumabe em pacientes hospitalizados com COVID-19 nos pacientes que estejam recebendo ou concluíram um curto período de tempo de corticosteroides, como dexametasona, sem evidência de infecção bacteriana ou viral (diferente do coronavírus da síndrome respiratória aguda grave 2 - SARS-CoV-2), que possa ser agravado pelo uso de tocilizumabe. Ainda, seu uso é recomendado nos pacientes que necessitam de oxigênio suplementar e nível de proteína C-reativa de 75mg/L ou mais, ou estão dentro de 48 horas após o início do oxigênio nasal de alto fluxo, pressão positiva contínua nas vias aéreas, VNI ou VMI (recomendação forte, certeza de evidência moderada).

- NIH: recomenda o uso de tocilizumabe em pacientes com COVID-19 que foram hospitalizados recentemente e apresentam necessidades crescentes de oxigênio e inflamação sistêmica (graduação BIIa).

- ERS: sugere o uso de terapia de anticorpo monoclonal antagonista do receptor de interleucina (IL) 6 para pacientes hospitalizados com COVID-19 que necessitem de oxigênio ou suporte ventilatório (recomendação condicional, certeza de evidência baixo). Além disso, sugere não oferecer terapia de anticorpo monoclonal antagonista do receptor de IL-6 para pacientes que não necessitem de oxigênio suplementar (recomendação condicional, certeza de evidência baixa).

Resumo das evidências

Na tomada de decisão, utilizamos as evidências apresentadas pela IDSA, datadas de 17 de fevereiro de 2021, que contemplam as evidências de forma atualizada, não tendo sido identificadas novas evidências relevantes que pudessem ter impacto na tomada de decisão. O perfil de evidências está apresentado na tabela 9S, correspondendo a pacientes hospitalizados com COVID-19.

Tabela 9S - Perfil de evidências: devemos utilizar tocilizumabe em pacientes hospitalizados com COVID-19?

Avaliação da certeza							Sumário de resultados				
Participantes nos estudos (n) Seguimento	Risco de viés	Inconsistência	Evidência indireta	Imprecisão	Viés de publicação	Qualidade geral da evidência	Taxas de eventos do estudo (%)		Efeito relativo (IC95%)	Efeitos absolutos potenciais	
							Com controle	Com tocilizumabe		Risco com controle	Diferença de risco com tocilizumabe
Mortalidade (seguimento: variação 28 dias para 30 dias)											
6.334 (8 ECRs)	Não grave*	Não grave	Não grave	Grave†	Nenhum	⊕⊕⊕◯ Moderada	893/3.054 (29,2%)	810/3.280 (24,7%)	RR = 0,91 (0,79 - 1,04)	292 por 1.000	26 menos por 1.000 (de 61 menos para 12 mais)
Deterioração clínica (seguimento: variação 14 dias para 30 dias)											
5.215 (7 ECRs)	Grave‡	Não grave	Não grave	Não grave§	Nenhum	⊕⊕⊕◯ Moderada	939/2.503 (37,5%)	799/2.712 (29,5%)	RR = 0,83 (0,77 - 0,89)	375 por 1.000	64 menos por 1.000 (de 86 menos para 41 menos)
Eventos adversos graves											
2.195 (7 ECRs)	Grave‡	Não grave	Não grave	Grave¶	Nenhum	⊕⊕◯◯ Baixa	141/946 (14,9%)	210/1.249 (16,8%)	RR = 0,89 (0,74 - 1,07)	149 por 1.000	16 menos por 1.000 (de 39 menos para 10 mais)

IC95% - intervalo de confiança de 95%; ECR - ensaio clínico randomizado; RR - risco relativo. * Apesar de alguns estudos não serem cegados para os participantes e investigadores, é improvável que afetem o desfecho mortalidade; † intervalo de confiança de 95% inclui tanto benefício quanto risco; ‡ em alguns estudos, faltou cegamento e, devido ao mecanismo de tocilizumabe (redução do marcador inflamatório), é provável que tenha ocorrido quebra de ocultação nos estudos cegos; § definição de deterioração clínica variou, com todos os estudos incluindo necessidade de ventilação e morte, mas outros estudos incluíram necessidade de admissão na unidade de terapia intensiva (dois estudos) ou relação entre pressão parcial de oxigênio e fração inspirada de oxigênio inferior a 150mmHg; ¶ intervalo de confiança inclui potencial risco e benefício. Ainda, poucos eventos relatados não atendem ao tamanho ótimo da informação e sugerem fragilidade na estimativa.

Referências

1. The REMAP-CAP Investigators, Gordon AC, Mouncey PR, Al-Beidh F, Rowan KM, Nichol AD, Arabi YM, et al. Interleukin-6 receptor antagonists in critically ill patients with Covid-19 - Preliminary report. medRxiv. 2021:2021.01.07.21249390[15]
2. Rosas IO, Bräu N, Waters M, Go RC, Hunter BD, Bhagani S, et al. Tocilizumab in hospitalized patients with COVID-19 pneumonia. medRxiv 2020:2020.08.27.20183442.[16]
3. Hermine O, Mariette X, Tharaux PL, Resche-Rigon M, Porcher R, Ravaud P; CORIMUNO-19 Collaborative Group. Effect of tocilizumab vs usual care in adults hospitalized with COVID-19 and moderate or severe pneumonia: a randomized clinical trial. JAMA Intern Med. 2021;181(1):32-40.[17]
4. Salama C, Han J, Yau L, Reiss WG, Kramer B, Neidhart JD, et al. Tocilizumab in patients hospitalized with Covid-19 pneumonia. N Engl J Med 2021; 384(1): 20-30.[18]
5. Salvarani C, Dolci G, Massari M, Merlo DF, Cavuto S, Savoldi L, Bruzzi P, Boni F, Braglia L, Turrà C, Ballerini PF, Sciascia R, Zammarchi L, Para O, Scotton PG, Inojosa WO, Ravagnani V, Salerno ND, Sainaghi PP, Brignone A, Codeluppi M, Teopompi E, Milesi M, Bertomoro P, Claudio N, Salio M, Falcone M, Cenderello G, Donghi L, Del Bono V, Colombelli PL, Angheben A, Passaro A, Secondo G, Pascale R, Piazza I, Facciolongo N, Costantini M; RCT-TCZ-COVID-19 Study Group. Effect of tocilizumab vs standard care on clinical worsening in patients hospitalized with COVID-19 pneumonia: a randomized clinical trial. JAMA Intern Med. 2020;181(1):24-31.[19]
6. Stone JH, Frigault MJ, Serling-Boyd NJ, Fernandes AD, Harvey L, Foulkes AS, Horick NK, Healy BC, Shah R, Bensaci AM, Woolley AE, Nikiforow S, Lin N, Sagar M, Schrager H, Huckins DS, Axelrod M, Pincus MD, Fleisher J, Sacks CA, Dougan M, North CM, Halvorsen YD, Thurber TK, Dagher Z, Scherer A, Wallwork RS, Kim AY, Schoenfeld S, Sen P, Neilan TG, Perugino CA, Unizony SH, Collier DS, Matza MA, Yinh JM, Bowman KA, Meyerowitz E, Zafar A, Drobni ZD, Bolster MB, Kohler M, D'Silva KM, Dau J, Lockwood MM, Cubbison C, Weber BN, Mansour MK; BACC Bay Tocilizumab Trial Investigators. Efficacy of tocilizumab in patients hospitalized with Covid-19. N Engl J Med. 2020;383(24):2333-44.[20]
7. Veiga VC, Prats JA, Farias DL, Rosa RG, Dourado LK, Zampieri FG, Machado FR, Lopes RD, Berwanger O, Azevedo LC, Avezum Á, Lisboa TC, Rojas SS, Coelho JC, Leite RT, Carvalho JC, Andrade LE, Sandes AF, Pintão MC, Castro CG Jr, Santos SV, de Almeida TM, Costa AN, Gebara OC, de Freitas FG, Pacheco ES, Machado DJ, Martin J, Conceição FG, Siqueira SR, Damiani LP, Ishihara LM, Schneider D, de Souza D, Cavalcanti AB, Scheinberg P; Coalition covid-19 Brazil VI Investigators. Effect of tocilizumab on clinical outcomes at 15 days in patients with severe or critical coronavirus disease 2019: randomised controlled trial. BMJ. 2021;372:n84.[21]
8. RECOVERY Collaborative Group. Tocilizumab in patients admitted to hospital with COVID-19 (RECOVERY): a randomised, controlled, open-label, platform trial. Lancet. 2021;397(10285):1637-45.[22]

Fonte: adaptada de Bhimraj A, Morgan RL, Shumaker AH, Lavergne V, Baden L, Cheng VC, Edwards KM, Gandhi R, Gallagher J, Muller WJ, O'Horo JC, Shoham S, Murad MH, Mustafa RA, Sultan S, Falck-Ytter Y. Infectious Diseases Society of America. Guidelines on the Treatment and Management of Patients with COVID-19: Infectious Diseases Society of America; 2021; Version 5.6.0. [cited 2021 May 10]. Available from: https://www.idsociety.org/practice-guideline/covid-19-guideline-treatment-and-management/ [5]

Foram identificados oito estudos clínicos.[15-21,23] Entre pacientes hospitalizados, o tocilizumabe apresentou tendência na redução de mortalidade aos 28 dias (RR de 0,91; IC95% 0,79 - 1,04; certeza de evidência moderada) e também demonstrou menor risco relativo para deterioração clínica, definida como morte, necessidade de VM, ECMO ou admissão na UTI (RR de 0,83; IC95% 0,77 - 0,89; certeza de evidência moderada), comparado a placebo ou aos cuidados usuais.

Dois estudos, RECOVERY e REMAP-CAP, contribuíram com maior peso na análise.[22,24] No RECOVERY, foram randomizados 4.116 participantes com hipóxia ($SatO_2$ < 92% ou em oxigenoterapia) e evidências de inflamação sistêmica (proteína C-reativa ≥ 75mg/L) para terapia usual ou terapia usual associada a tocilizumabe intravenoso (primeira dose de 400 a 800mg, ajustada para o peso; uma segunda dose poderia ser administrada após 12 - 24 horas, caso as condições do paciente não melhorassem). A idade dos pacientes foi 64 ± 14 anos, 45% não estavam recebendo suporte respiratório, exceto oxigenoterapia simples (nove pacientes não estavam recebendo oxigênio na randomização), 41% estavam em suporte respiratório não invasivo (incluindo oxigênio nasal de alto fluxo, pressão positiva contínua nas vias aéreas e VNI), e 14% estavam em VMI. O uso do tocilizumabe esteve associado a uma menor taxa de mortalidade (31% *versus* 35%; RR de 0,85; IC95% 0,76 - 0,94) e à maior probabilidade de alta hospitalar vivo em 28 dias (57% *versus* 50%; RR de 1,22; IC95% 1,12 - 1,33), quando comparado aos cuidados usuais. Entre os pacientes que não estavam em VMI no início do estudo, o uso de tocilizumabe foi associado a um menor risco de progressão para VM ou óbito (35% *versus* 42%; RR de 0,84; IC95% 0,77 - 0,92), em comparação aos cuidados usuais. O risco de evolução para óbito ou uso de VM também foi menor nos pacientes que receberam tocilizumabe e estavam sem uso de suporte ventilatório no início do estudo (RR de 0,82; IC95% 0,69 - 0,96), assim como nos que estavam em uso de VNI (RR de 0,89; IC95% 0,80 - 0,98), quando comparados aos casos em cuidados usuais. O tocilizumabe reduziu o risco de óbito em 28 dias tanto no grupo sem suporte ventilatório (RR de 0,81; IC95% 0,67 - 0,99) quanto em VNI (RR de 0,86; IC95% 0,74 - 1,00) e, por outro lado, não reduziu a mortalidade entre os pacientes em VMI (RR de 0,93; IC95% 0,74 - 1,18) comparado aos cuidados usuais. O benefício foi observado apenas nos pacientes que estavam em uso de corticosteroides.

O estudo REMAP-CAP avaliou os efeitos do tocilizumabe (8mg/kg, com limite de 800mg, intravenoso; a dose poderia ser repetida caso o paciente apresentasse melhora insuficiente) e do sarilumabe (400mg, intravenoso) em pacientes críticos com COVID-19, que estivessem recebendo terapia de suporte respiratório ou cardiovascular há, no máximo, 24 horas na UTI. Foram randomizados 895 pacientes, e o desfecho principal do estudo foi o total de dias livres de suporte respiratório e cardiovascular, sendo calculado o número de dias sem suporte até o dia 21 dia de internação, ou seja, um resultado maior representa uma recuperação mais rápida. A idade dos pacientes foi 61 ± 13 anos, e todos os pacientes (exceto três) estavam recebendo suporte respiratório no momento da randomização, sendo 29% em oxigênio por CNAF, 42% em VNI e 29% em VM. O número médio de dias sem suporte respiratório ou cardiovascular foi de 10 (intervalo interquartil -1 - 16) no Grupo Tocilizumabe, 11 (intervalo interquartil 0 - 16) no Grupo Sarilumabe e zero (intervalo interquartil -1 - 15) no Grupo Controle. A RC ajustada para sobrevida intra-hospitalar foi de 1,64 (intervalo de credibilidade de 95% 1,14 - 2,35) para tocilizumabe e 2,01 (intervalo de credibilidade de 95% 1,18 - 4,71) para sarilumabe, comparadas ao grupo controle. A RC ajustada para sobrevida nos pacientes que estavam em VM foi 1,87 (intervalo de credibilidade de 95% 1,26 - 2,81) e naqueles em VM foi de 1,51 (intervalo de credibilidade de 95% 0,88 - 2,63).

Questão 5 - Devemos utilizar cloroquina ou hidroxicloroquina em pacientes hospitalizados com COVID-19?

Recomendação 5.1 - recomendamos não utilizar cloroquina ou hidroxicloroquina em pacientes hospitalizados com COVID-19 (recomendação forte, certeza da evidência moderada).

Recomendações de outras diretrizes

Das diretrizes consideradas, sete fizeram recomendações referentes ao uso de hidroxicloroquina e cloroquina. Nenhuma diretriz recomendou o uso desses medicamentos no tratamento dos pacientes com COVID-19:

- *Australian Taskforce:* recomenda não utilizar hidroxicloroquina no tratamento de pacientes com COVID-19 (recomendação forte, certeza da evidência alta).

- IDSA: recomenda não utilizar hidroxicloroquina em pacientes hospitalizados com COVID-19 (recomendação forte, certeza da evidência moderada). Além disso, recomenda não utilizar hidroxicloroquina em associação com azitromicina (recomendação forte, certeza da evidência baixa).

- Diretrizes brasileiras (AMIB, SBI e SBPT): sugerem não utilizar hidroxicloroquina ou cloroquina de rotina no tratamento de pacientes com COVID-19 (recomendação condicional, certeza da evidência baixa).

- SSC: recomenda não utilizar hidroxicloroquina em pacientes adultos com COVID-19 grave ou crítica (recomendação forte, certeza da evidência moderada).

- WHO: recomenda não utilizar hidroxicloroquina ou cloroquina no tratamento de pacientes com COVID-19 (recomendação forte, certeza da evidência moderada).

- NIH: recomenda não utilizar hidroxicloroquina ou cloroquina (associadas ou não ao uso de azitromicina) no tratamento de pacientes hospitalizados com COVID-19 (graduação AI). Além disso, recomenda não utilizar hidroxicloroquina de alta dose no tratamento de pacientes com COVID-19 (graduação AI).

- ERS: recomenda não utilizar hidroxicloroquina em pacientes com COVID-19 (hospitalizados ou não; recomendação forte, certeza da evidência moderada). Além disso, recomenda não utilizar hidroxicloroquina em associação com azitromicina no tratamento de pacientes com COVID-19 (recomendação condicional, certeza da evidência moderada).

Resumo das evidências

Na tomada de decisão, utilizamos as evidências apresentadas pela IDSA, datadas de 23 de dezembro de 2020, que contemplam as evidências de forma atualizada, não tendo sido identificadas novas evidências relevantes que pudessem ter impacto na tomada de decisão. O perfil de evidências está apresentado na tabela 10S, correspondendo a pacientes hospitalizados com COVID-19.

Cinco ensaios clínicos randomizados apresentaram tendência de mortalidade em pacientes tratados com hidroxicloroquina em comparação aos que não fizeram uso do medicamento (RR de 1,08; IC95% 0,99 - 1,19).[25,26,28]

Um ensaio clínico randomizado identificou que pacientes tratados com hidroxicloroquina apresentaram mediana de tempo de internação maior em comparação com pacientes não tratados (mediana de 16 dias *versus* 13 dias) e menor probabilidade de receberem alta com vida no 28º dia do estudo (RR de 0,92; IC95% 0,85 - 0,99).[29]

Em relação à segurança, considerando o conjunto de evidências, o tratamento com hidroxicloroquina pode aumentar o risco de efeitos adversos (RR de 2,36; IC95% 1,49 - 3,75) e de efeitos adversos graves (RC ajustada de 1,26; IC95% 0,56 - 2,84).[26,31-33] Um ensaio clínico randomizado e dois estudos não randomizados demonstraram aumento do risco de prolongamento do intervalo QT nos pacientes tratados com hidroxicloroquina em comparação aos pacientes não tratados (RR de 8,47; IC95% 1,14 - 63,03; e RR de 2,89; IC95% 1,62 - 5,16).[26,30,34] Por fim, um estudo observou percentual maior de pacientes que tiveram arritmias no grupo que fez uso de hidroxicloroquina em comparação ao grupo de pacientes que não utilizaram o medicamento (16% *versus* 10%; RR de 1,56; IC95% 0,97 - 2,50).[35]

Questão 6 - Devemos utilizar azitromicina em pacientes hospitalizados com COVID-19?

Recomendação 6.1 - recomendamos não utilizar azitromicina, associada ou não à cloroquina ou à hidroxicloroquina, em pacientes hospitalizados com COVID-19 (recomendação forte, certeza da evidência moderada).

Recomendações de outras diretrizes

Das diretrizes consideradas, cinco fizeram recomendações referentes ao uso de azitromicina, em associação com hidroxicloroquina ou não. Nenhuma diretriz recomendou o uso desses medicamentos no tratamento dos pacientes com COVID-19:

***- Australian Taskforce*:** não recomenda o uso de azitromicina no tratamento de pacientes com COVID-19 (recomendação forte, certeza de evidência baixa).

- IDSA: recomenda não utilizar hidroxicloroquina em associação com azitromicina em pacientes com COVID-19 (recomendação forte, certeza da evidência baixa).

- Diretrizes brasileiras (AMIB, SBI e SBPT): sugerem não utilizar a combinação de hidroxicloroquina ou cloroquina e azitromicina de rotina no tratamento de pacientes com COVID-19 (recomendação condicional, certeza da evidência muito baixa).

- NIH: recomenda não utilizar a combinação de hidroxicloroquina ou cloroquina e azitromicina no tratamento de pacientes hospitalizados com COVID-19 (graduação AI).

Tabela 10S - Perfil de evidências: devemos utilizar cloroquina ou hidroxicloroquina em pacientes hospitalizados com COVID-19?

Avaliação da certeza							Sumário de resultados				
							Taxas de eventos do estudo (%)			Efeitos absolutos potenciais	
Participantes nos estudos (n) Seguimento	Risco de viés	Inconsistência	Evidência indireta	Imprecisão	Viés de publicação	Avaliação da certeza	Com controle	Com hidroxicloroquina	Efeito relativo (IC95%)	Risco com controle	Diferença de risco com hidroxicloroquina
Mortalidade (seguimento: variação 22 dias para 49 dias)											
7.508 (5 ECRs)	Não grave*	Não grave	Não grave†	Grave‡	Nenhum	⨁⨁⨁◯ Moderada	908/4.532 (20,0%)	561/2.976 (18,9%)	RR = 1,08 (0,99 - 1,19)	200 por 1.000	16 mais por 1.000 (de 2 menos para 38 mais)
Estado clínico (avaliado com: Escala de 7 pontos; mais alto significa piora da gravidade)											
332 (1 ECR)	Grave§	Não grave	Não grave	Grave¶	Nenhum	⨁⨁◯◯ Baixa	173	159	-	A média do estado clínico foi 0	DM 1.21 mais alto (0.69 mais alto para 2.11 mais alto)
Progressão para ventilação mecânica invasiva											
5.609 (2 ECRs)	Grave||	Não grave	Não grave	Grave‡	Nenhum	⨁⨁◯◯ Baixa	281/3.447 (8,2%)	193/2.162 (8,9%)	RR = 1,10 (0,92 - 1,31)	82 por 1.000	8 mais por 1.000 (de 7 menos para 25 mais)
Arritimias											
492 (1 estudo observacional)	Muito grave#	Não grave	Não grave	Muito grave¶	Nenhum	⨁◯◯◯ Muito baixa	23/221 (10,4%)	44/271 (16,2%)	RR = 1,56 (0,97 - 2,50)	104 por 1.000	58 mais por 1.000 (de 3 menos para 156 mais)
Eventos adversos											
491 (4 ECRs)	Grave**	Não grave	Não grave	Grave¶	Nenhum	⨁⨁◯◯ Baixa	18/176 (10,2%)	94/315 (29,8%)	RR = 2,36 (1,49 - 3,75)	102 por 1.000	139 mais por 1.000 (de 50 mais para 281 mais)
Eventos adversos graves (avaliado com: evento médico adverso que leva à morte, uma experiência com risco de vida, prolongamento da hospitalização ou deficiência ou incapacidade persistente ou significativa)											
479 (1 ECR)	Não grave	Não grave	Não grave	Muito grave¶	Nenhum	⨁⨁◯◯ Baixa	11/237 (4,6%)	14/242 (5,8%)	RR =1,26 (0,56 - 2,84)	46 por 1.000	11 mais por 1.000 (de 20 menos para 75 mais)
Prolongamento QT (ECR)											
147 (1 ECR)	Não grave	Não grave	Não grave	Muito grave||	Nenhum	⨁⨁◯◯ Baixa	1/58 (1,7%)	13/89 (14,6%)	RR = 8,47 (1,14 - 63,03)	17 por 1.000	129 mais por 1.000 (de 2 mais para 1.000 mais)
Prolongamento QT (EC não randomizado)											
666 (2 estudos observacionais)	Muito grave#††	Não grave	Não grave	Grave[f]	Nenhum	⨁◯◯◯ Muito baixa	13/311 (4,2%)	46/355 (13,0%)	RR = 2,89 (1,62 - 5,16)	42 por 1.000	79 mais por 1.000 (de 26 mais para 174 mais)

IC95% - intervalo de confiança de 95%; ECR - ensaio clínico randomizado; RR - risco relativo; DM - diferença da média; RC - razão de chances. * Cointervenções foram fornecidas aos pacientes em ambos os estudos, mas equilibradas entre os braços; † Cavalcanti et al. 2020 excluíram pessoas que recebem oxigênio suplementar a uma taxa de mais de 4L/minuto; ‡ intervalo de confiança de 95% não pode excluir o potencial risco ou benefício; § Cavalcanti foi um ensaio clínico aberto; ¶ intervalo de confiança de 95% inclui o potencial risco e benefício. Poucos eventos sugerem o potencial fragilidade na estimativa; | | poucos eventos sugerem potencial fragilidade na estimativa; # preocupações com confusão não avaliada e residual. Múltiplas cointervenções recebidas entre os braços; ** não relataram sobre cegamento (incluindo comitê de adjudicação de resultados), geração de sequência ou ocultação de alocação; Chen J 2020: todos os pacientes receberam alfa interferon nebulizado; 80% versus 67,7% dos indivíduos receberam Abidiol no braço da hidroxicloroquina versus placebo, respectivamente. Dois indivíduos no braço de controle receberam lopinavir/ ritonavir; †† Mahevas et al. 2020 não relatam eventos adversos no braço de comparação.

Referências

1. RECOVERY Collaborative Group, Horby P, Mafham M, Linsell L, Bell JL, Staplin N, Emberson JR, et al. Effect of hydroxychloroquine in hospitalized patients with COVID-19. N Engl J Med. 2020;383(21):2030-40.[25]
2. Cavalcanti AB, Zampieri FG, Rosa RG, Azevedo LC, Veiga VC, Avezum A, Damiani LP, Marcadenti A, Kawano-Dourado L, Lisboa T, Junqueira DL, de Barros E Silva PG, Tramujas L, Abreu-Silva EO, Laranjeira LN, Soares AT, Echenique LS, Pereira AJ, Freitas FG, Gebara OC, Dantas VC, Furtado RH, Milan EP, Golin NA, Cardoso FF, Maia IS, Hoffmann Filho CR, Kormann AP, Amazonas RB, Bocchi de Oliveira MF, Serpa-Neto A, Falavigna M, Lopes RD, Machado FR, Berwanger O; Coalition Covid-19 Brazil I Investigators. Hydroxychloroquine with or without azithromycin in mild-to-moderate Covid-19. N Engl J Med. 2020;383(21):2041-52.[26]
3. WHO Solidarity Trial Consortium, Pan H, Peto R, Henao-Restrepo AM, Preziosi MP, Sathiyamoorthy V, Abdool Karim Q, et al. Repurposed antiviral drugs for Covid-19 - interim WHO solidarity trial results. N Engl J Med. 2021;384(6):497-511.[27]
4. Self WH, Semler MW, Leither LM, Casey JD, Angus DC, Brower RG, et al. Effect of hydroxychloroquine on clinical status at 14 days in hospitalized patients with COVID-19: a randomized clinical trial. JAMA. 2020;324(21):2165-76.(28)
5. Ulrich RJ, Troxel AB, Carmody E, Eapen J, Bäcker M, DeHovitz JA, et al. Treating COVID-19 with hydroxychloroquine (TEACH): a multicenter, double-blind randomized controlled trial in hospitalized patients. Open Forum Infect Dis. 2020;7(10):ofaa446.[29]
6. Rosenberg ES, Dufort EM, Udo T, Wilberschied LA, Kumar J, Tesoriero J, et al. Association of treatment with hydroxychloroquine or azithromycin with in-hospital mortality in patients with COVID-19 in New York state. JAMA; 2020;323(24):2493-502.[30]
7. Chen J, Liu D, Liu L, Liu P, Xu Q, Xia L, et al. [A pilot study of hydroxychloroquine in treatment of patients with moderate COVID-19]. Zhejiang Da Xue Xue Bao Yi Xue Ban. 2020;49(2):215-9. Chinese.[31]
8. Chen Z, Hu J, Zhang Z, Jiang S, Han S, Yan D, et al. Efficacy of hydroxychloroquine in patients with COVID-19: results of a randomized clinical trial. medRxiv 2020:2020.03.22.20040758.[32]
9. Tang W, Cao Z, Han M, Wang Z, Chen J, Sun W, et al. Hydroxychloroquine in patients with mainly mild to moderate coronavirus disease 2019: open label, randomised controlled trial. BMJ. 2020;369:m1849.[33]
10. Mahévas M, Tran VT, Roumier M, Chabrol A, Paule R, Guillaud C, et al. No evidence of clinical efficacy of hydroxychloroquine in patients hospitalised for COVID-19 infection and requiring oxygen: results of a study using routinely collected data to emulate a target trial. medRxiv 2020:2020.04.10.20060699.[34]

Fonte: adaptada de Bhimraj A, Morgan RL, Shumaker AH, Lavergne V, Baden L, Cheng VC, Edwards KM, Gandhi R, Gallagher J, Muller WJ, O'Horo JC, Shoham S, Murad MH, Mustafa RA, Sultan S, Falck-Ytter Y. Infectious Diseases Society of America. Guidelines on the Treatment and Management of Patients with COVID-19: Infectious Diseases Society of America; 2021; Version 5.6.0. [cited 2021 May 10]. Available from: https://www.idsociety.org/practice-guideline/covid-19-guideline-treatment-and-management/[5]

- ERS: recomenda não utilizar hidroxicloroquina em associação com azitromicina no tratamento de pacientes com COVID-19 (recomendação condicional, certeza da evidência moderada).

Resumo das evidências

Na tomada de decisão, utilizamos as evidências apresentadas pela IDSA, datadas de 23 de dezembro de 2020, que contemplam as evidências de forma atualizada, não tendo sido identificadas novas evidências relevantes que pudessem ter impacto na tomada de decisão. O perfil de evidências está apresentado na tabela 11S, correspondendo a pacientes hospitalizados com COVID-19.

Um ensaio clínico randomizado não descartou o risco de mortalidade intra-hospitalar em pacientes tratados com uma combinação de hidroxicloroquina e azitromicina (*hazard ratio* - HR - 0,64; IC95% 0,18 - 2,21).[28] Três estudos não randomizados não identificaram associação entre tratamento com a combinação de hidroxicloroquina e azitromicina e mortalidade (Ip et al., 2020: HR de 0,98; IC95% 0,75 - 1,28; Magagnoli et al., 2020: HR ajustada de 0,89; IC95% 0,45 - 1,77; Rosenberg et al., 2020: HR ajustada de 1,35; IC95% 0,79 - 2,40).[30,35,36] Ensaio clínico randomizado realizado no Brasil com 447 pacientes graves ou críticos hospitalizados por COVID-19, avaliando a associação de hidroxicloroquina e azitromicina, comparada a hidroxicloroquina, não identificou diferença significativa na melhora clínica com a associação dos dois medicamentos, com o grupo controle possuindo resultados absolutos mais favoráveis (RC de 1,36; IC95% 0,94 - 1,97; valor de p = 0,11).[39]

Em relação à segurança do medicamento, um ensaio clínico randomizado demonstrou aumento do risco de prolongamento do intervalo QT em pacientes tratados com combinação de hidroxicloroquina e azitromicina (risco relativo – RR – de 8,50; IC95% 1,16-62,31).[29] Além disso, dois estudos reportaram prolongamento do intervalo QT em 10 dos 95 pacientes avaliados, demonstrando risco elevado para arritmias clinicamente relevantes em pacientes que fizeram uso da combinação de hidroxicloroquina e azitromicina.[39,40] Por fim, um estudo de caso-controle que avaliou pacientes com COVID-19 tratados com a combinação de hidroxicloroquina e azitromicina (n = 22) em comparação a pacientes saudáveis (n = 34) identificou valores maiores mínimos (415 *versus* 376 milissegundos), médios (453 *versus* 407 milissegundos) e máximos (533 *versus* 452 milissegundos) de intervalo QTc.[41]

Questão 7 - Devemos utilizar casirivimabe + imdevimabe em pacientes hospitalizados com COVID-19?

Recomendação 7.1 - sugerimos não utilizar casirivimabe + imdevimabe em pacientes hospitalizados com COVID-19 (recomendação condicional, certeza da evidência muito baixa).

Recomendações de outras diretrizes

Das diretrizes avaliadas, nenhuma avaliou o uso de casirivimabe + imdevimabe em pacientes hospitalizados. A diretriz do NIH realizou recomendação para pacientes não hospitalizados com COVID-19 leve a moderada:

- NIH: recomenda o uso de bamlanivimabe 700mg + etesevimabe 1.400mg; ou casirivimabe 1.200mg + imdevimabe 1.200mg em pacientes não hospitalizados com COVID-19 leve a moderada (graduação AIII).

Resumo das evidências

Não foram identificados estudos clínicos publicados avaliando casirivimabe + imdevimabe em pacientes hospitalizados. Até o momento há dados publicados mostrando redução de carga viral em pacientes ambulatoriais, contudo não há dados avaliando pacientes hospitalizados.

Questão 8 - Devemos utilizar rendesivir em pacientes hospitalizados com COVID-19?

Recomendação 8.1 - sugerimos não utilizar rendesivir em pacientes hospitalizados com COVID-19 (recomendação condicional, certeza da evidência baixa).

Recomendações de outras diretrizes

Das diretrizes consideradas, sete avaliaram o rendesivir, e cinco delas recomendam ou sugerem o uso de rendesivir em alguns perfis de pacientes hospitalizados com COVID-19:

Tabela 11S - Perfil de evidências: devemos utilizar azitromicina em pacientes hospitalizados com COVID-19?

Avaliação da certeza							Sumário de resultados				
							Taxas de eventos do estudo (%)			Efeitos absolutos potenciais	
Participantes nos estudos (n) Seguimento	Risco de viés	Inconsistência	Evidência indireta	Imprecisão	Viés de publicação	Avaliação da certeza	Com controle	Com hidroxicloroquina e azitromicina	Efeito relativo (IC95%)	Risco com controle	Diferença de risco com hidroxicloroquina e azitromicina
Mortalidade (ECR; seguimento: variação 22 dias para 49 dias)											
345 (1 ECR)	Não grave*	Não grave	Não grave†	Grave‡§¶	Nenhum	⊕⊕⊕◯ Moderada	6/173 (3,5%)	5/172 (2,9%)	HR = 0,64 (0,18 - 2,21)	35 por 1.000	12 menos por 1.000 (de 28 menos para 40 mais)
Mortalidade (ensaio clínico não randomizado)											
(3 estudos observacionais)	Muito grave||	Não grave	Não grave	Grave§	Nenhum	⊕◯◯◯ Muito baixa	Três estudos não randomizados não conseguiram identificar uma associação entre pessoas tratadas com HCQ + AZ e mortalidade: Ip et al.relataram uma razão de risco (HR - *hazard ratio*) ajustada de 0,98 (IC95% 0,75-1,28); Magagnoli relatou uma HR ajustada em um subconjunto após o ajuste do escore de propensão de 0,89 (IC95% 0,45-1,77); Rosenberg et al.2020 relataram HR de 1,35 (IC95% 0,79-2,40) (Ip et al., Magagnoli et al. 2020, Rosenberg et al.2020)					
Estado clínico (avaliado com: escala de 7 pontos, valores mais altos representam piores desfechos clínicos)											
345 (1 ECR)	Não grave¶#	Não grave	Não grave†	Grave§**	Nenhum	⊕⊕⊕◯ Moderada	173	172	-	A média do estado clínico foi 0	DM 0,99 mais alto (0,57 mais alto para 1,73 mais alto)
Falha virológica (seguimento: variação 5 dias para 6 dias; avaliado com teste PCR)											
83 (2 estudos observacionais)	Muito grave††	Grave‡‡	Grave§§	Grave‡	Nenhum	⊕◯◯◯ Muito baixa	12/12 (100,0%)	29/71 (40,8%)	Não estimável	1.000 por 1.000	
Prolongamento QT (ECR)											
174 (1 ECR)	Não grave	Não grave	Grave§§¶¶	Grave‡	Nenhum	⊕⊕◯◯ baixa	1/58 (1,7%)	17/116 (14,7%)	RR = 8,50 (1,16 - 62,31)	17 por 1.000	129 mais por 1.000 (de 3 mais para 1.000 mais)
Prolongamento QT (ensaio clínico não randomizado)											
95 (2 estudos observacionais)	Muito grave††	Não grave	Grave¶¶	Grave‡	Nenhum	⊕◯◯◯ Muito baixa		10/95 (10,5%)	Não estimável	0 por 1.000	
Eventos adversos graves											
289 (1 ECR)	Grave**	Não grave	Não grave	Grave‡§	Nenhum	⊕⊕◯◯ Baixa	0/50 (0,0%)	5/239 (2,1%)	RR = 2,34 (0,13 - 41,61)	0:1 por 000	0 menos por 1.000 (de 0 menos para 0 menos)

IC95% - intervalo de confiança de 95%; ECR - ensaio clínico randomizado; HR - hazard ratio; HCQ hidroxicloroquinha; AZ – azitromicina; RR - risco relativo; DM - diferença da média; PCR – proteína C-reativa. Evidência adicional considerada para tomada de decisão: ensaio clínico randomizado realizado no Brasil com 447 pacientes graves ou críticos hospitalizados por COVID-19, avaliando a associação de hidroxicloroquina e azitromicina, comparado à hidroxicloroquina, não identificou diferença significativa na melhora clínica com a associação dos dois medicamentos, com o Grupo Controle possuindo resultados absolutos melhores (razão de chance de 1,36; intervalo de confiança de 95% 0,94-1,97; valor de p = 0,11). * As cointervenções foram fornecidas aos pacientes, mas equilibradas entre os braços. Cavalcanti 2020 foi um estudo aberto; no entanto, provavelmente não influenciou o resultado da mortalidade; †Cavalcanti et al. 2020 excluíram pessoas que recebem oxigênio suplementar a uma taxa de mais de 4L/minuto; ‡ um número muito pequeno de eventos. Tamanho ótimo da informação não atingido; § o intervalo de confiança de 95% inclui potencial risco e benefício; ¶ o grupo elaborador revisou as evidências e considerou como grave, ao invés de muito grave, a imprecisão para o desfecho mortalidade e como não grave em vez de grave, para risco de viés no desfecho estado clínico, assim a qualidade da evidência foi julgada como moderada e não baixa para esses desfechos, divergindo da avaliação inicial realizada pela Infectious Diseases Society of America; || preocupações com confusão não avaliada e residual. Múltiplas cointervenções recebidas entre os braços; # Cavalcanti et al. realizaram um ensaio clínico aberto; ** tamanho ótimo da informação não atingido; †† nenhum grupo de controle contemporâneo; nenhum ajuste para a gravidade da linha de base, resultando em alto risco de confusão residual; ‡‡ duas séries de casos da França mostraram resultados divergentes; §§ marcador substituto para mortalidade ou resolução da COVID-19; ¶¶ avaliação indireta de mortalidade específica por arritmia; ## a azitromicina e a hidroxicloroquina podem causar de forma independente o prolongamento do intervalo QT. Usadas juntas, pode haver um efeito aditivo. Deve-se ter cuidado com outros agentes conhecidos por prolongar o intervalo QT.

Referências

1. Cavalcanti AB, Zampieri FG, Rosa RG, Azevedo LC, Veiga VC, Avezum A, Damiani LP, Marcadenti A, Kawano-Dourado L, Lisboa T, Junqueira DL, de Barros E Silva PG, Tramujas L, Abreu-Silva EO, Laranjeira LN, Soares AT, Echenique LS, Pereira AJ, Freitas FG, Gebara OC, Dantas VC, Furtado RH, Milan EP, Golin NA, Cardoso FF, Maia IS, Hoffmann Filho CR, Kormann AP, Amazonas RB, Bocchi de Oliveira MF, Serpa-Neto A, Falavigna M, Lopes RD, Machado FR, Berwanger O; Coalition Covid-19 Brazil I Investigators. Hydroxychloroquine with or without azithromycin in mild-to-moderate Covid-19. N Engl J Med. 2020;383(21):2041-52.[26]
2. Rosenberg ES, Dufort EM, Udo T, Wilberschied LA, Kumar J, Tesoriero J, et al. Association of treatment with hydroxychloroquine or azithromycin with in-hospital mortality in patients with COVID-19 in New York State. JAMA. 2020;323(24):2493-502.[30]
3. Magagnoli J, Narendran S, Pereira F, Cummings TH, Hardin JW, Sutton SS, et al. Outcomes of hydroxychloroquine usage in United States veterans hospitalized with Covid-19. Med (N Y). 2020;1(1):114-127.e3.[35]
4. Ip A, Berry DA, Hansen E, Goy AH, Pecora AL, Sinclaire BA, et al. Hydroxychloroquine and tocilizumab therapy in COVID-19 patients-An observational study. PLoS One. 2020;15(8):e0237693.[36]
5. Gautret P, Lagier JC, Parola P, Hoang VT, Meddeb L, Mailhe M, et al. Hydroxychloroquine and azithromycin as a treatment of COVID-19: results of an open-label non-randomized clinical trial. Int J Antimicrob Agents. 2020;56(1):105949.[37]
6. Gautret P, Lagier JC, Parola P, et al. Clinical and microbiological effect of a combination of hydroxychloroquine and azithromycin in 80 COVID-19 patients with at least a six-day follow up: a pilot observational study. Travel Med Infect Dis. 2020;34:101663.[38]
7. Molina JM, Delaugerre C, Le Goff J, Mela-Lima B, Ponscarme D, Goldwirt L, et al. No evidence of rapid antiviral clearance or clinical benefit with the combination of hydroxychloroquine and azithromycin in patients with severe COVID-19 infection. Med Mal Infect. 2020;50(4):384.[39]
8. Chorin E, Dai M, Shulman E, Wadhwani L, Roi-Bar-Cohen, Barbhaiya C, et al. The QT interval in patients with SARS-CoV-2 infection treated with hydroxychloroquine/azithromycin. medRxiv. 2020:2020.04.02.20047050.[40]

Fonte: adaptada de Bhimraj A, Morgan RL, Shumaker AH, Lavergne V, Baden L, Cheng VC, Edwards KM, Gandhi R, Gallagher J, Muller WJ, O'Horo JC, Shoham S, Murad MH, Mustafa RA, Sultan S, Falck-Ytter Y. Infectious Diseases Society of America. Guidelines on the Treatment and Management of Patients with COVID-19: Infectious Diseases Society of America; 2021; Version 5.6.0. [cited 2021 May 10]. Available from: https://www.idsociety.org/practice-guideline/covid-19-guideline-treatment-and-management/[5]

- *Australian Taskforce*: sugere o uso de rendesivir para o tratamento de adultos hospitalizados com COVID-19 moderada a grave que não requerem ventilação (recomendação condicional, certeza da evidência moderada). Recomenda não iniciar rendesivir para o tratamento de adultos hospitalizados com COVID-19 em ventilação (recomendação forte, certeza da evidência baixa).

- IDSA: sugere o uso de rendesivir em pacientes hospitalizados com COVID-19 grave (recomendação condicional, certeza da evidência moderada). Sugere o uso de rendesivir por 5 dias, ao invés de 10 dias, em pacientes hospitalizados com COVID-19 em uso de oxigênio suplementar, mas que não estejam em VM ou ECMO (recomendação condicional, certeza da evidência baixa). Recomenda não usar rendesivir como rotina em pacientes com COVID-19 admitidos no hospital sem uso de oxigênio suplementar e SaO_2 > 94% em ar ambiente (recomendação condicional, certeza da evidência muito baixa).

- NICE: sugere utilizar rendesivir por 5 dias para tratamento de pneumonia em pacientes que estejam hospitalizados e em uso de oxigênio suplementar, mas que não estejam em VMI (recomendação condicional, certeza da evidência moderada). Recomenda não usar rendesivir para tratamento de pneumonia em pacientes hospitalizados com COVID-19 e que estejam em uso de VMI, com exceção do cenário de pesquisa (recomendação forte, certeza da evidência moderada).

- SSC: sugere usar rendesivir intravenoso em pacientes adultos com COVID-19 grave que não estejam em VM (recomendação fraca, certeza de evidência moderada). Sugere não usar rendesivir intravenoso em pacientes críticos com COVID-19 que estejam em VM (recomendação fraca, certeza de evidência baixa).

- WHO: sugere não usar rendesivir em pacientes hospitalizados com COVID-19, independente da severidade da doença (recomendação condicional, certeza da evidência baixa).

- NIH: não há dados suficientes para recomendar ou não recomendar o rendesivir em pacientes hospitalizados que não requerem uso de oxigênio. O uso de rendesivir pode ser apropriado em pacientes que apresentam um alto risco de progressão da doença que não requerem uso de oxigênio. Recomenda o uso de rendesivir para pacientes hospitalizados com COVID-19 que requerem oxigênio suplementar mínimo, mas que não requerem uso de dispositivo de alto fluxo, VNI, VMI ou ECMO (graduação BIIa).

- ERS: os autores se posicionaram por não recomendar o uso de rendesivir em pacientes hospitalizados com COVID-19 e que não requerem VMI (sem recomendação, certeza da evidência moderada).

Resumo das evidências

Na tomada de decisão, utilizamos as evidências apresentadas pela IDSA, datadas de 8 de abril de 2021, que contemplam as evidências de forma atualizada, não tendo sido identificadas novas evidências relevantes que pudessem ter impacto na tomada de decisão. O perfil de evidências está apresentado na tabela 12S, correspondendo a pacientes hospitalizados com COVID-19.

Três ensaios clínicos randomizados avaliaram a efetividade do tratamento com rendesivir em pacientes hospitalizados, em uso de oxigênio suplementar, ou com saturação < 94% em ar ambiente.[27,42,43] Não houve impacto na mortalidade com o uso de rendesivir (13,5% *versus* 14,4%; RR de 0,92; IC95% 0,77 - 1,10). Estudo único mostrou redução no tempo até recuperação (RR de 1,31; IC95% 1,12 - 1,52) e necessidade de VM (12,9% *versus* 22,5%; RR de 0,57; IC95% 0,42 - 0,79). Redução na necessidade de VM não foi observada no estudo SOLIDARITY.

O estudo SOLIDARITY randomizou 5.475 pacientes internados com COVID-19 para tratamento com rendesivir ou placebo, não demonstrando benefício no uso de rendesivir para o desfecho de mortalidade (RR de 0,95; IC95% 0,81 - 1,11). As análises de subgrupos foram consistentes com o resultado geral, demonstrando ausência de benefício no tratamento com rendesivir para os pacientes que não estavam em VM (RR 0,86; IC95% 0,67 - 1,11), assim como nos que estavam em uso de suporte ventilatório (RR 1,20; IC95% 0,80 - 1,80) no início do estudo. Os autores também publicaram metanálise que incluiu quatro ensaios clínicos randomizados, corroborando a ausência de benefício com o uso de rendesivir para o desfecho de mortalidade (RR de 0,91; IC95% 0,79 - 1,05).[45] Na metanálise, para o subgrupo de pacientes inicialmente sem ventilação, houve tendência para benefício, mas sem obter diferença estatisticamente significativa. A avaliação completa sobre o uso do rendesivir para o tratamento de COVID-19 está disponível em nota técnica publicada pelo Ministério da Saúde.[46]

Tabela 12S - Perfil de evidências: devemos utilizar rendesivir em pacientes hospitalizados com COVID-19?

Avaliação da certeza							Sumário de resultados				
Participantes nos estudos (n) Seguimento	Risco de viés	Inconsistência	Evidência indireta	Imprecisão	Viés de publicação	Qualidade geral da evidência	Taxas de eventos do estudo (%) Com controle	Taxas de eventos do estudo (%) Com rendesevir	Efeito relativo (IC95%)	Efeitos absolutos potenciais Risco com controle	Efeitos absolutos potenciais Diferença de risco com rendesevir
Mortalidade (seguimento: variação 28 dias para 29 dias)											
5.319 (3 ECRs)	Grave*†‡	Não grave	Não grave	Grave§	Nenhum	⊕⊕◯◯ Baixa	374/2.593 (14,4%)	369/2.726 (13,5%)	RR = 0,92 (0,77 - 1,10)	144 por 1.000	12 menos por 1.000 (de 33 menos para 14 mais)
Tempo para recuperação (seguimento: média 29 dias)											
957 (1 ECR)	Grave‡	Não grave	Não grave	Não grave	Nenhum	⊕⊕⊕◯ Moderada	306/471	345/486	RR = 1,31 (1,12 - 1,52)	650 por 1.000	97 mais por 1.000 pacientes por ano (de 41 mais para 147 mais)
Melhora clínica (seguimento: média 28 dias)											
236 (1 ECR)	Grave*†	Não grave	Não grave	Grave‡	Nenhum	⊕⊕◯◯ Baixa	45/78 (57,7%)	103/158 (65,2%)	RR = 1,13 (0,91 - 1,41)	577 por 1.000	75 mais por 1.000 (de 52 menos para 237 mais)
Necessidade de ventilação mecânica (seguimento: média 29 dias)											
766 (1 ECR)	Não grave	Não grave	Não grave	Grave¶	Nenhum	⊕⊕⊕◯ Moderada	82/364 (22,5%)	52/402 (12,9%)	RR = 0,57 (0,42 - 0,79)	225 por 1.000	97 menos por 1.000 (de 131 menos para 47 menos)
Eventos adversos graves											
1.177 (2 ECRs)	Não grave	Não grave	Não grave	Grave||	Nenhum	⊕⊕⊕◯ Moderada	53/545 (9,7%)	44/632 (7,0%)	RR = 0,88 (0,74 - 1,06)	97 por 1.000	12 menos por 1.000 (de 25 menos para 6 mais)
Hospitalização											
236 (1 ECR)	Não grave*†	Não grave	Não grave	Muito grave§	Nenhum	⊕⊕◯◯ Baixa	78	158	-	A média de hospitalização foi 0	DM 1 mais alto (0,12 mais alto para 1,88 mais alto)
Duração da ventilação mecânica											
236 (1 ECR)	Não grave*†	Não grave	Não grave	Grave§	Nenhum	⊕⊕⊕◯ Moderada	78	158	-	A média duração da ventilação mecânica foi 0	DM 8,5 mais alto (9,14 mais alto para 7,86 mais alto)

IC95% - intervalo de confiança de 95%; ECR - ensaio clínico randomizado; RR - risco relativo; DM - diferença da média. Foi considerado o estudo SOLIDARITY, que não observou redução na necessidade de ventilação mecânica para tomada de decisão. * As cointervenções recebidas em Wang et al. 2020 incluem: interferon alfa-2b, lopinavir/ritonavir, vasopressores, antibióticos e corticoterapia. Elas foram equilibradas entre os braços; † Wang et al. 2020 finalizaram mais cedo devido à falta de recrutamento. Ensaio iniciado após a redução na apresentação de novos pacientes (a maioria dos pacientes inscritos posteriormente à doença); ‡ a análise post-hoc de pacientes com doença grave de Pan et al. 2020 e Beigel et al. 2020 pode introduzir viés; § intervalo de confiança de 95% pode não incluir um efeito clinicamente significativo; ¶ poucos eventos não atendem ao tamanho ótimo da informação e sugerem fragilidade na estimativa; | | intervalo de confiança de 95% não pode excluir o potencial risco ou benefício. Além disso, poucos eventos não atendem ao tamanho ótimo de informação.

Referências
1. Wang Y, Zhang D, Du G, Du R, Zhao J, Jin Y, et al. Remdesivir in adults with severe COVID-19: a randomised, double-blind, placebo-controlled, multicentre trial. Lancet. 2020;395(10236):1569-78.[41]
2. Beigel JH, Tomashek KM, Dodd LE, Mehta AK, Zingman BS, Kalil AC, Hohmann E, Chu HY, Luetkemeyer A, Kline S, Lopez de Castilla D, Finberg RW, Dierberg K, Tapson V, Hsieh L, Patterson TF, Paredes R, Sweeney DA, Short WR, Touloumi G, Lye DC, Ohmagari N, Oh MD, Ruiz-Palacios GM, Benfield T, Fätkenheuer G, Kortepeter MG, Atmar RL, Creech CB, Lundgren J, Babiker AG, Pett S, Neaton JD, Burgess TH, Bonnett T, Green M, Makowski M, Osinusi A, Nayak S, Lane HC; ACTT-1 Study Group Members. Remdesivir for the treatment of Covid-19 - Final report. N Engl J Med. 2020;383(19):1813-26.[42]
3. WHO Solidarity Trial Consortium, Pan H, Peto R, Henao-Restrepo AM, Preziosi MP, Sathiyamoorthy V, Abdool Karim Q, et al. Repurposed antiviral drugs for Covid-19 - interim WHO solidarity trial results. N Engl J Med. 2021;384(6):497-511.[27]
Fonte: adaptada de Bhimraj A, Morgan RL, Shumaker AH, Lavergne V, Baden L, Cheng VC, Edwards KM, Gandhi R, Gallagher J, Muller WJ, O'Horo JC, Shoham S, Murad MH, Mustafa RA, Sultan S, Falck-Ytter Y. Infectious Diseases Society of America. Guidelines on the Treatment and Management of Patients with COVID-19: Infectious Diseases Society of America; 2021; Version 5.6.0. [cited 2021 May 10]. Available from: https://www.idsociety.org/practice-guideline/covid-19-guideline-treatment-and-management/[5]

Questão 9 - Devemos utilizar plasma convalescente em pacientes hospitalizados com COVID-19?

Recomendação 9.1 - recomendamos não utilizar plasma convalescente em pacientes hospitalizados com COVID-19 (recomendação forte, certeza da evidência moderada).

Recomendações de outras diretrizes

As quatro diretrizes consideradas não recomendam o uso de plasma convalescente em pacientes hospitalizados com COVID-19:

- *Australian Taskforce*: recomenda não utilizar plasma convalescente em pacientes com COVID-19 (recomendação forte, certeza da evidência moderada).

- IDSA: sugere não utilizar plasma convalescente em pacientes hospitalizados com COVID-19 (recomendação condicional, certeza da evidência baixa).

- SSC: sugere não utilizar plasma convalescente fora dos ensaios clínicos (recomendação condicional, certeza da evidência baixa).

- NIH: não há dados suficientes para recomendar a favor ou contra o uso de plasma convalescente de alto título para o tratamento de COVID-19 em pacientes imunossuprimidos. Não recomenda utilizar plasma convalescente em pacientes hospitalizados com COVID-19 em VM que não têm imunidade prejudicada (graduação AI).

Resumo das evidências

Na tomada de decisão, utilizamos as evidências apresentadas pela IDSA, datadas de 7 de abril de 2021, que contemplam as evidências de forma atualizada, não tendo sido identificadas novas evidências relevantes que pudessem ter impacto na tomada de decisão. O perfil de evidências está apresentado na tabela 13S, correspondendo a pacientes hospitalizados com COVID-19.

Foram encontrados 11 ensaios clínicos randomizados e um registro de estudo de braço único avaliando os desfechos mortalidade, uso de VM, eventos adversos graves e eventos adversos.[44-47,55] Dez ensaios clínicos randomizaram 13.026 pacientes hospitalizados com COVID-19 para receber transfusão com plasma convalescente, e um avaliou seu uso a nível ambulatorial.[51] A transfusão de plasma convalescente não mostrou impacto na mortalidade (RR de 0,86; IC95% 0,69 - 1,06) e não reduziu a necessidade de VM (RR de 1,11; IC95% 0,95 - 1,30). A maioria dos pacientes avaliados foi oriunda do estudo RECOVERY, o qual identificou taxas idênticas de mortalidade (24% *versus* 24%; RR de 1,00; IC95% 0,93 - 1,07), alta hospitalar vivo em 28 dias (66% *versus* 67%; RR de 0,98; IC95% 0,94 - 1,03) e morte ou evolução para VM em pacientes que não estavam em VM na linha de base (28% *versus* 29%; RR de 0,99; IC95% 0,93 - 1,05).[52]

No maior estudo sobre segurança (n = 20.000), dentro de 4 horas após a conclusão da transfusão de plasma convalescente, os autores relataram 146 eventos adversos graves classificados como reações transfusionais (< 1%).[53] No prazo de 7 dias após a transfusão, foram relatadas 1.711 mortes (taxa de mortalidade de 8,56%; IC95% 8,18 - 8,95). Além disso, foram relatados 1.136 eventos adversos graves: 643 eventos cardíacos (569 julgados como não relacionados à transfusão), 406 eventos hipotensivos sustentados que necessitam suporte pressórico intravenoso e 87 eventos tromboembólicos ou trombóticos (55 julgados como não relacionados à transfusão). Quatro estudos entre pacientes hospitalizados por COVID-19 não puderam excluir um aumento em eventos adversos leves a graves entre pacientes que receberam plasma convalescente (RR de 1,02; IC95% 0,64 - 1,62).[46,47,49,51]

Questão 10 - Devemos utilizar ivermectina em pacientes hospitalizados com COVID-19?

Recomendação 10.1 - sugerimos não utilizar ivermectina em pacientes hospitalizados com COVID-19 (recomendação condicional, certeza da evidência muito baixa).

Recomendações de outras diretrizes

Das diretrizes consideradas, três recomendaram não utilizar ivermectina e uma diretriz não julgou devido a dados insuficientes:

- *Australian Taskforce*: recomenda não utilizar ivermectina no tratamento de pacientes com COVID-19 fora de estudos randomizados com aprovação ética apropriada (recomendação forte, certeza da evidência muito baixa).

Tabela 13S - Perfil de evidências: devemos utilizar plasma convalescente em pacientes hospitalizados com COVID-19?

Avaliação da certeza							Sumário de resultados				
							Taxas de eventos do estudo (%)			Efeitos absolutos potenciais	
Participantes nos estudos (n) Seguimento	Risco de viés	Inconsistência	Evidência indireta	Imprecisão	Viés de publicação	Qualidade geral da evidência	Com não usar plasma convalescente	Com plasma convalescente	Efeito relativo (IC95%)	Risco com controle	Diferença de risco com plasma convalescente
Mortalidade (ECR; seguimento: variação 15 dias para 60 dias)											
13.186 (10 ECRs)	Não grave*†	Não grave	Não grave	Grave‡	Nenhum	⊕⊕⊕◯ Moderada	1.518/6.477 (23,4%)	1.508/6.709 (22,5%)	RR = 0,86 (0,69 - 1,06)	234 por 1.000	33 menos por 1.000 (de 73 menos para 14 mais)
Ventilação mecânica											
990 (3 ECRs)	Grave§	Não grave	Não grave	Grave¶	Nenhum	⊕⊕◯◯ Baixa	161/440 (36,6%)	181/550 (32,9%)	RR = 1,11 (0,95 - 1,30)	366 por 1.000	40 mais por 1.000 (de 18 menos para 110 mais)
EAG (sobrecarga circulatória associada à transfusão, lesão pulmonar aguda relacionada à transfusão, reação alérgica transfusional grave)											
(1 estudo observacional)	Crítico||	Não grave	Não grave	Não grave	Nenhum	⊕◯◯◯ Muito baixa	EAG de 20.000 pacientes transfundidos: nas primeiras 4 horas, dos EAGs, 63 mortes foram relatadas (0,3% de todas as transfusões), e 13 dessas mortes foram julgadas como possivelmente ou provavelmente relacionadas à transfusão de plasma convalescente. Houve 83 EAGs não relacionados a morte, com 37 notificações de eventos associados à transfusão com sobrecarga circulatória, 20 relatos de lesão pulmonar aguda relacionada à transfusão e 26 relatos de reação alérgica transfusional grave				
EAGs (mortalidade, eventos cardíacos, trombóticos, hipotensivos sustentados que requerem intervenção; seguimento: média 7 dias)											
(1 estudo observacional)	Crítico||	Não grave	Não grave	Não grave	Nenhum	⊕◯◯◯ Muito baixa	EAGs de 20.000 pacientes transfundidos: dentro de 7 dias após a transfusão, 1.711 mortes (8,56%) e 1.136 eventos adversos graves (5,68%) foram relatadas. EAGs de não mortalidade incluíram: 643 eventos cardíacos (569 julgados como não relacionados à transfusão); 406 eventos hipotensivos sustentados que necessitaram suporte pressórico intravenoso; e 87 eventos tromboembólicos ou trombóticos (55 julgados como não relacionados à transfusão)				
Qualquer evento adverso (ECRs)											
673 (4 ECRs)	Grave§	Não grave	Não grave	Grave#	Nenhum	⊕⊕◯◯ Baixa	52/240 (21,7%)	102/433 (23,6%)	RR = 1,02 (0,64 - 1,62)	217 por 1.000	4 mais por 1.000 (de 78 menos para 134 mais)

IC95% - intervalo de confiança de 95%; ECR - ensaio clínico randomizado; RR - risco relativo; EAG - eventos adversos graves.* Li et al. 2020: o tempo entre o início dos sintomas e a randomização foi de mais de 14 dias para > 90% (mediana de 30 dias), nenhum ajuste para cointervenções, métodos de ocultação de alocação não relatados e participantes e profissionais de saúde não cegados; † muitos estudos apresentaram preocupações por serem abertos, com ocultação de alocação não relatada e nenhum ajuste para cointervenções; ‡ intervalo de confiança de 95% inclui o benefício apreciável; no entanto, não pode excluir o potencial para nenhum efeito; § as preocupações com o estudo incluem ser um estudo aberto e a avaliação dos desfechos; ¶ intervalo de confiança de 95% pode não incluiu uma redução clinicamente significativa na necessidade de ventilação mecânica; || nenhum efeito comparativo disponível. Alguma subjetividade na classificação dos desfechos como sendo relacionados à transfusão; # intervalo de confiança de 95% inclui potencial tanto de risco como benefício. N pequeno de eventos.

Referências

1. Li L, Zhang W, Hu Y, Tong X, Zheng S, Yang J, et al. Effect of convalescent plasma therapy on time to clinical improvement in patients with severe and life-threatening COVID-19: a randomized clinical trial. JAMA. 2020;324(5):460-70.[44]
2. Gharbharan A, Jordans CC, Geurtsvankessel C, den Hollander JG, Karim F, Mollema FP, et al. Convalescent plasma for COVID-19. A randomized clinical trial. medRxiv. 2020:2020.07.01.20139857.[45]
3. AlQahtani M, Abdulrahman A, Almadani A, Alali SY, Al Zamrooni AM, Hejab AH, et al. Randomized controlled trial of convalescent plasma therapy against standard therapy in patients with severe COVID-19 disease. Sci Rep. 2021;11(1):9927.[46]
4. Avendaño-Solà C, Ramos-Martínez A, Muñez-Rubio E, Ruiz-Antorán B, de Molina RM, Torres F, et al. Convalescent plasma for COVID-19: a multicenter, randomized clinical trial. medRxiv. 2020:2020.08.26.20182444.[47]
5. Ray Y, Paul SR, Bandopadhyay P, D'Rozario R, Sarif J, Lahiri A, et al. Clinical and immunological benefits of convalescent plasma therapy in severe COVID-19: insights from a single center open label randomised control trial. medRxiv 2020.11.25.20237883.[48]
6. Simonovich VA, Burgos Pratx LD, Scibona P, Beruto MV, Vallone MG, Vázquez C, et al. A randomized trial of convalescent plasma in Covid-19 severe pneumonia. N Engl J Med. 2021;384(7):619-29.[49]
7. Agarwal A, Mukherjee A, Kumar G, Chatterjee P, Bhatnagar T, Malhotra P; PLACID Trial Collaborators. Convalescent plasma in the management of moderate covid-19 in adults in India: open label phase II multicentre randomised controlled trial (PLACID Trial). BMJ. 2020;371:m3939.[50]
8. O'Donnell MR, Grinsztejn B, Cummings MJ, Justman JE, Lamb MR, Eckhardt CM, et al. A randomized, double-blind controlled trial of convalescent plasma in adults with severe COVID-19. J Clin Invest. 2021;131(13):e150646.[51]
9. RECOVERY Collaborative Group. Convalescent plasma in patients admitted to hospital with COVID-19 (RECOVERY): a randomised, controlled, open-label, platform trial. Lancet. 2021;397(10289):2049-59.[52]
10. Balcells ME, Rojas L, Le Corre N, Martínez-Valdebenito C, Ceballos ME, Ferrés M, et al. Early versus deferred anti-SARS-CoV-2 convalescent plasma in patients admitted for COVID-19: a randomized phase II clinical trial. PLoS Med. 2021;18(3):e1003415.[53]
11. Joyner MJ, Bruno KA, Klassen SA, Kunze KL, Johnson PW, Lesser ER, et al. Safety update: COVID-19 convalescent plasma in 20,000 hospitalized patients. Mayo Clin Proc. 2020;95(9):1888-97.[54]

Fonte: adaptada de Bhimraj A, Morgan RL, Shumaker AH, Lavergne V, Baden L, Cheng VC, Edwards KM, Gandhi R, Gallagher J, Muller WJ, O'Horo JC, Shoham S, Murad MH, Mustafa RA, Sultan S, Falck-Ytter Y. Infectious Diseases Society of America. Guidelines on the Treatment and Management of Patients with COVID-19: Infectious Diseases Society of America; 2021; Version 5.6.0. [cited 2021 May 10]. Available from: https://www.idsociety.org/practice-guideline/covid-19-guideline-treatment-and-management/[5]

- IDSA: sugere não utilizar ivermectina no tratamento de pacientes com COVID-19 fora do contexto de ensaios clínicos (recomendação condicional, certeza da evidência muito baixa).
- WHO: recomenda não utilizar ivermectina no tratamento de pacientes com COVID-19 fora do contexto de ensaios clínicos (recomendação forte, certeza da evidência muito baixa).
- NIH: não há dados suficientes para que o painel recomende a favor ou contra o uso de ivermectina para o tratamento de COVID-19.

Resumo das evidências

Na tomada de decisão, utilizamos as evidências apresentadas pela IDSA, datadas de 12 de fevereiro de 2021, que contemplam as evidências de forma atualizada, não tendo sido identificadas novas evidências relevantes que pudessem ter impacto na tomada de decisão. O perfil de evidências está apresentado na tabela 14S, correspondendo a pacientes hospitalizados com COVID-19.

Quatro estudos observacionais[56-59] sugerem que a ivermectina pode reduzir a mortalidade em pacientes com COVID-19 (RR de 0,57; IC95% 0,36 - 0,90), um ensaio clínico randomizado apresenta tendência de aumento da resolução de sintomas (RR de 1,07; IC95% 0,69 - 1,65)[60] e três ensaios clínicos randomizados apresentam tendência de depuração viral (RR de 1,33; IC95% 1,00 - 1,78).[59,61,62] Ainda, não se pode excluir o potencial risco de eventos adversos entre pessoas hospitalizadas com COVID-19 tratadas com ivermectina (RR de 0,88; IC95% 0,47 - 1,63).[59]

Questão 11 - Devemos utilizar colchicina em pacientes hospitalizados COM COVID-19?

Recomendação 11.1 - recomendamos não utilizar colchicina em pacientes hospitalizados com COVID-19 (recomendação forte, certeza da evidência moderada).

Recomendações de outras diretrizes

Das diretrizes consideradas, três recomendaram não utilizar colchicina em pacientes hospitalizados:
- *Australian Taskforce*: recomenda não utilizar colchicina no tratamento de pacientes com COVID-19 fora de estudos randomizados com aprovação ética apropriada (recomendação forte, certeza da evidência muito baixa).
- NIH: recomenda não utilizar colchicina em pacientes hospitalizados para tratamento de COVID-19 fora do contexto de ensaios clínicos (graduação AIII).
- ERS: recomenda não utilizar colchicina em pacientes hospitalizados com COVID-19 (recomendação condicional, certeza da evidência muito baixa).

Resumo das evidências

Na tomada de decisão, utilizamos as evidências apresentadas pela ERS, publicadas em abril de 2021, que contemplam as evidências de forma atualizada, não tendo sido identificadas novas evidências relevantes que pudessem ter impacto na tomada de decisão. O perfil de evidências está apresentado na tabela 15S, correspondendo a pacientes hospitalizados com COVID-19.

Um ensaio clínico randomizado encontrou menor risco de deterioração, porém foi baseado em um pequeno número de eventos e, portanto, é de natureza incerta.[63] Outros desfechos importantes, como admissão à UTI (RC de 1,06; IC95% 0,06 - 18,45) e mortalidade (RR de 0,66; IC95% 0,24 - 1,85), não foram significativamente reduzidos com a terapia.[63-65] O estudo RECOVERY randomizou pacientes hospitalizados com COVID-19 para receber colchicina ou cuidado usual. Em avaliação de 11.162 pacientes, não houve diferença significativa no desfecho primário de mortalidade em 28 dias entre os braços. Houve 2.178 mortes, sendo 20% no braço da colchicina e 19% no braço de tratamento convencional (RR de 1,02; IC95% 0,94 - 1,11; valor de p = 0,63), sendo o estudo precocemente interrompido por futilidade.[65] Além disso, um aumento significativo nos eventos adversos (principalmente diarreia) foi observado com a administração de colchicina (RC de 3,96; IC95% 1,72 - 9,12).[63,64]

Tabela 14S - Perfil de evidências: devemos utilizar ivermectina em pacientes hospitalizados com COVID-19?

Avaliação da certeza							Sumário de resultados				
							Taxas de eventos do estudo (%)			Efeitos absolutos potenciais	
Participantes nos estudos (n) Seguimento	Risco de viés	Inconsistência	Evidência indireta	Imprecisão	Viés de publicação	Avaliação da certeza	Com controle	Com ivermectina	Efeito relativo (IC95%)	Risco com controle	Diferença de risco com ivermectina
Mortalidade (ensaio clínico não randomizado)											
531 (4 estudos observacionais)	Grave*	Não grave	Não grave	Grave†	Nenhum	⊕◯◯◯ Muito baixa	35/260 (13,5%)	28/271 (10,3%)	RR = 0,57 (0,36 - 0,90)	135 por 1.000	58 menos por 1.000 (de 86 menos para 13 menos)
Resolução de sintomas (seguimento: 7 dias)											
50 (1 ECR)	Grave‡	Não grave	Não grave	Muito grave†	Nenhum	⊕◯◯◯ Muito baixa	15/25 (60,0%)	16/25 (64,0%)	RR = 1,07 (0,69 - 1,65)	600 por 1.000	42 mais por 1.000 (de 186 menos para 390 mais)
Clearance viral no dia 7 (ECR) (seguimento: variação 7 dias para 29 dias)											
109 (3 ECRs)	Grave§	Não grave	Grave¶	Muito grave†	Nenhum	⊕◯◯◯ Muito baixa	22/55 (40,0%)	29/54 (53,7%)	RR = 1,33 (1,00 - 1,78)	400 por 1.000	132 mais por 1.000 (de 0 menos para 312 mais)
Eventos adversos (seguimento: 28 dias)											
24 (1 ECR)	Não grave	Não grave	Não grave	Muito grave†	Nenhum	⊕⊕◯◯ Baixa	8/12 (66,7%)	7/12 (58,3%)	RR = 0,88 (0,47 - 1,63)	667 por 1.000	80 menos por 1.000 (de 353 menos para 420 mais)

IC95% - intervalo de confiança de 95%; RR - risco relativo; ECR - ensaio clínico randomizado. * Preocupações em relação à confusão não medida e residual. Gorial et al. 2020: estudo de braço único com controle histórico. Hashim et al. 2020 usaram dias pares e ímpares para alocar os indivíduos nos grupos com pacientes críticos não incluídos no grupo placebo. Rajtal et al., os corticosteroides foram usados em 19,6% dos pacientes em tratamento usual versus 39,8% dos pacientes com ivermectina; † intervalo de confiança de 95% inclui o potencial de benefício apreciável, bem como o potencial de dano. Poucos eventos relatados não atendem ao tamanho ideal da informação e sugerem fragilidade na estimativa; ‡ ensaio clínico aberto pode levar a viés na medição de desfechos subjetivos. § Podder et al. 2020 atribuíram participantes com base em números de registro pares ou ímpares. Além disso, 20 pacientes foram excluídos após a randomização sem análise de sensibilidade para explorar desequilíbrio entre os braços de tratamento; ¶ o clearance viral é um desfecho substituto para a melhora clínica, como hospitalização, necessidade de cuidados na unidade de terapia intensiva e ventilação mecânica.

Referências

1. Gorial FI, Mashhadani S, Sayaly HM, Dakhil BD, AlMashhadani MM, Aljabory AM, et al. Effectiveness of Ivermectin as add-on Therapy in COVID-19 Management (Pilot Trial). medRxiv 2020:2020.07.07.20145979.[56]
2. Hashim HA, Maulood MF, Rasheed AM, Fatak DF, Kabah KK, Abdulamir AS. Controlled randomized clinical trial on using ivermectin with doxycycline for treating COVID-19 patients in Baghdad, Iraq. medRxiv 2020:2020.10.26.20219345.[57]
3. Rajter JC, Sherman MS, Fatteh N, Vogel F, Sacks J, Rajter JJ. Use of ivermectin is associated with lower mortality in hospitalized patients with coronavirus disease 2019: the ivermectin in COVID nineteen study. Chest. 2021;159(1):85-92.[58]
4. Chaccour C, Casellas A, Blanco-Di Matteo A, Pineda I, Fernandez-Montero A, Ruiz-Castillo P, et al. The effect of early treatment with ivermectin on viral load, symptoms and humoral response in patients with non-severe COVID-19: a pilot, double-blind, placebo-controlled, randomized clinical trial. EClinicalMedicine. 2021;32:100720.[59]
5. Chachar AZ, Khan KA, Asif M, Khushbakht, Khaqan A, Basri R. Effectiveness of ivermectin in SARS-CoV-2/COVID-19 patients. Int J Sci. 2020;9:31-5.[60]
6. Ahmed S, Karim MM, Ross AG, Hossain MS, Clemens JD, Sumiya MK, et al. A five-day course of ivermectin for the treatment of COVID-19 may reduce the duration of illness. Int J Infect Dis. 2021;103:214-6.[61]
7. Podder CS, Chowdhury N, Sina MI, Haque W. Outcome of ivermectin treated mild to moderate COVID-19 cases: a single-centre, open-label, randomised controlled study. IMC J Med Sci. 2020;14(2):002.[62]
Fonte: adaptada de Bhimraj A, Morgan RL, Shumaker AH, Lavergne V, Baden L, Cheng VC, Edwards KM, Gandhi R, Gallagher J, Muller WJ, O'Horo JC, Shoham S, Murad MH, Mustafa RA, Sultan S, Falck-Ytter Y. Infectious Diseases Society of America. Guidelines on the Treatment and Management of Patients with COVID-19: Infectious Diseases Society of America; 2021; Version 5.6.0. [cited 2021 May 10]. Available from: https://www.idsociety.org/practice-guideline/covid-19-guideline-treatment-and-management/[5]

Tabela 15S - Perfil de evidências: devemos utilizar colchicina em pacientes hospitalizados com COVID-19?

Avaliação da certeza							Sumário de resultados				
Participantes nos estudos (n) Seguimento	Risco de viés	Inconsistência	Evidência indireta	Imprecisão	Viés de publicação	Qualidade geral da evidência	Taxas de eventos do estudo (%) Com controle	Com colchicina	Efeito relativo (IC95%)	Efeitos absolutos potenciais Risco com controle	Diferença de risco com colchicina
Deterioração (definido como piora de 2 pontos na escala ordinal da OMS)											
105 (1 ECR)	Grave*†‡	Não grave	Não grave	Não grave	Nenhum	⊕⊕⊕◯ Moderada	7/50 (14,0%)	1/55 (1,8%)	RC = 0,11 (0,01 - 0,96)	140 por 1.000	122 menos por 1.000 (de 138 menos para 5 menos)
Mortalidade											
140 (2 ECRs)	Grave†	Não grave	Não grave	Grave‡	Nenhum	⊕⊕◯◯ Baixa	1196/5816 (0,6%)	1174/5701 (0,6%)	RR = 0,21 (0,24 - 1,85)	206 por 1.000	70 menos por 1.000 (de 156 menos para 175 mais)
Admissão em UTI											
35 (1 ECR)	Grave†	Não grave	Não grave	Muito grave‡	Nenhum	⊕◯◯◯ Muito baixa	1/18 (5,6%)	1/17 (5,9%)	RC = 1,06 (0,06 - 18,45)	56 por 1.000	3 mais por 1.000 (de 52 menos para 465 mais)
Eventos adversos - diarreia											
140 (2 ECRs)	Grave†	Não grave	Não grave	Não grave	Nenhum	⊕⊕⊕◯ Moderada	10/68 (14,7%)	29/72 (40,3%)	RC = 3,96 (1,72 - 9,12)	147 por 1.000	259 mais por 1.000 (de 82 mais para 464 mais)

IC95% - intervalo de confiança de 95%; OMS - Organização Mundial da Saúde; ECR - ensaio clínico randomizado; RC - razão de chances; RR - risco relativo. Para a tomada de decisão foi considerado o estudo RECOVERY (comunicado de imprensa), publicado após a diretriz da *European Respiratory Society*. *Um único centro, estudo aberto, relato subótimo de desfechos; † relato subótimo de desfecho, um estudo possuía múltiplos desfechos principais sem controle para múltiplas comparações estatísticas; ‡ intervalo de confiança de 95%amplo, que inclui tanto benefício como malefício.

Referências

1. Deftereos SG, Giannopoulos G, Vrachatis DA, Siasos GD, Giotaki SG, Gargalianos P, Metallidis S, Sianos G, Baltagiannis S, Panagopoulos P, Dolianitis K, Randou E, Syrigos K, Kotanidou A, Koulouris NG, Milionis H, Sipsas N, Gogos C, Tsoukalas G, Olympios CD, Tsagalou E, Migdalis I, Gerakari S, Angelidis C, Alexopoulos D, Davlouros P, Hahalis G, Kanonidis I, Katritsis D, Kolettis T, Manolis AS, Michalis L, Naka KK, Pyrgakis VN, Toutouzas KP, Triposkiadis F, Tsioufis K, Vavouranakis E, Martinèz-Dolz L, Reimers B, Stefanini GG, Cleman M, Goudevenos J, Tsiodras S, Tousoulis D, Iliodromitis E, Mehran R, Dangas G, Stefanadis C; GRECCO-19 investigators. Effect of colchicine vs standard care on cardiac and inflammatory biomarkers and clinical outcomes in patients hospitalized with coronavirus disease 2019: the GRECCO-19 randomized clinical trial. JAMA Netw Open. 2020;3(6):e2013136.[63]
2. Lopes MI, Bonjorno LP, Giannini MC, Amaral NB, Menezes PI, Dib SM, et al. Beneficial effects of colchicine for moderate to severe COVID-19: a randomised, double-blinded, placebo controlled clinical trial. RMD Open. 2021;7(1):e001455.[64]
3. RECOVERY Collaborative Group. Colchicine in patients admitted to hospital with COVID-19 (RECOVERY): a randomised, controlled, open-label, platform trial. Lancet Respir Med. 2021;9(12):1419-26.[65]

Fonte: adaptada de Chalmers JD, Crichton ML, Goeminne PC, Cao B, Humbert M, Shteinberg M, et al. Management of hospitalised adults with coronavirus disease-19 (COVID-19): A European Respiratory Society living guideline. Eur Respir J. 2021;57(4):2100048.[66]

Questão 12 - Devemos utilizar lopinavir/ritonavir em pacientes hospitalizados com covid-19?

Recomendação 12.1 - recomendamos não utilizar lopinavir/ritonavir em pacientes hospitalizados com COVID-19 (recomendação forte, certeza da evidência moderada).

Recomendações de outras diretrizes

Das oito diretrizes consideradas, seis recomendaram não utilizar lopinavir/ritonavir em pacientes hospitalizados:

- *Australian Taskforce*: recomenda não utilizar lopinavir/ritonavir no tratamento de pacientes com COVID-19 (recomendação forte, certeza da evidência moderada).

- IDSA: recomenda não utilizar a combinação de lopinavir/ritonavir em pacientes hospitalizados com COVID-19 (recomendação forte, certeza da evidência moderada).

- Diretrizes brasileiras (AMIB, SBI e SBPT): sugere não utilizar lopinavir/ritonavir de rotina no tratamento da COVID-19 (recomendação fraca, certeza de evidência baixo).

- WHO: recomenda não utilizar lopinavir/ritonavir para tratamento de pacientes com COVID-19 (recomendação forte, certeza de evidência moderada).

- NIH: recomenda não utilizar lopinavir/ritonavir em pacientes hospitalizados para tratamento de COVID-19 (graduação AI).

- ERS: recomenda não utilizar lopinavir/ritonavir em pacientes hospitalizados com COVID-19 (recomendação forte, certeza da evidência baixa).

Resumo das evidências

Na tomada de decisão, utilizamos as evidências apresentadas pela IDSA, datadas de 22 de novembro de 2020, que contemplam as evidências de forma atualizada, não tendo sido identificadas novas evidências relevantes que pudessem ter impacto na tomada de decisão. O perfil de evidências está apresentado na tabela 16S, correspondendo a pacientes hospitalizados com COVID-19.

Entre os pacientes hospitalizados com COVID-19, o tratamento com lopinavir/ritonavir falhou em mostrar ou excluir um efeito benéfico na mortalidade (RR de 1,00; IC95% 0,89 - 1,13)(27,67,68) ou necessidade de VMI (RR de 1,12; IC95% 0,93 - 1,34).(67,68) Da mesma forma, lopinavir/ritonavir pode reduzir a falha na melhora clínica em 14 dias, porém o resultado é incerto (RR de 0,78; IC95% 0,63 - 0,97).(67) Ainda, no estudo de Cao et al., 14% dos pacientes em tratamento com lopinavir/ritonavir foram incapazes de completar o curso de administração em 14 dias.(67)

Tabela 16S - Perfil de evidências: devemos utilizar lopinavir/ritonavir em pacientes hospitalizados com COVID-19?

Certeza da evidência							Sumário de Resultados				
Participantes (estudos) Seguimento	Risco de viés	Inconsistência	Evidência indireta	Imprecisão	Viés de publicação	Certeza da evidência	Taxas de eventos do estudo (%) Com controle	Com lopinavir/ritonavir	Efeito relativo (IC95%)	Potenciais efeitos absolutos Risco com controle	Diferença de risco com lopinavir/ritonavir
Mortalidade (seguimento: média 28 dias)											
8.007 (3 ECRs)	Não grave*	Não grave	Não grave	Grave†	Nenhum	⊕⊕⊕◯ Moderada‡	938/4.896 (19,2%)	538/3.111 (17,3%)	RR = 1,00 (0,89 - 1,13)	192 por 1.000	0 menos por 1.000 (de 21 menos para 25 mais)
Ventilação mecânica invasiva (seguimento: média 28 dias)											
5.035 (2 ECRs)	Grave*§	Não grave	Não grave	Grave†	Nenhum	⊕⊕◯◯ Baixa	297/3.380 (8,8%)	166/1.655 (10,0%)	RR = 1,12 (0,93 - 1,34)	88 por 1.000	11 mais por 1.000 (de 6 menos para 30 mais)
Efeitos adversos que levaram a descontinuação do tratamento											
(1 ECR)	Grave*	Não grave	Não grave	Muito grave¶	Nenhum	⊕◯◯◯ Muito baixa	Quase 14% dos pacientes que receberam lopinavir-ritonavir foram incapazes de completar o curso de administração de 14 dias. Isso foi devido principalmente a eventos adversos gastrintestinais, incluindo anorexia, náusea, desconforto abdominal ou diarreia, bem como dois eventos adversos graves, ambos gastrite aguda. Dois receptores tiveram erupções cutâneas autolimitadas. Esses efeitos colaterais, incluindo os riscos de lesão hepática, pancreatite, erupções cutâneas mais graves e prolongamento do intervalo QT, e o potencial para múltiplas interações medicamentosas devido à inibição do CYP3A, estão bem documentados com essa combinação de medicamentos. O perfil de efeitos colaterais observado no estudo atual desperta preocupação sobre o uso de regimes de dosagem de lopinavir-ritonavir maiores ou mais prolongados em esforços para melhorar os resultados				
Falha na melhora clínica aos 14 dias (seguimento: média 14 dias)											
199 (1 ECR)	Grave*	Não grave	Não grave	Muito grave||	Nenhum	⊕◯◯◯ Muito baixa	70/100 (70,0%)	54/99 (54,5%)	RR = 0,78 (0,62 - 0,97)	700:1.000	154 menos por 1.000 (de 266 menos para 21 menos)

IC95% - intervalo de confiança de 95%; ECR - ensaio clínico randomizado; RR - risco relativo. * Estudos não cegados, os quais podem afetar os desfechos que exigem julgamento, como a forma como os investigadores julgam a melhora clínica ou decidem interromper o tratamento em pacientes com efeitos colaterais; † intervalo de confiança de 95% pode não incluir uma diferença significativa; ‡ intenção de tratar modificada para esse desfecho segundo Cao et al. 2020; algumas mortes foram excluídas quando o medicamento não foi administrado; § um paciente randomizado para o braço lopinavir-ritonavir em Cao et al. 2020 foi utilizou ventilação mecânica no início do estudo; ¶ pequeno número de eventos tornando as estimativas altamente incertas; || o limite superior do intervalo de confiança de 95% cruza o limite de melhora significativa, pois a estimativa do pior caso é uma redução do risco relativo de 3%.

Referências

1. Cao B, Wang Y, Wen D, Liu W, Wang J, Fan G, et al. A trial of lopinavir-ritonavir in adults hospitalized with severe Covid-19. N Engl J Med. 2020;382(19):1787-99.[67]
2. WHO Solidarity Trial Consortium, Pan H, Peto R, Henao-Restrepo AM, Preziosi MP, Sathiyamoorthy V, Abdool Karim Q, et al. Repurposed antiviral drugs for Covid-19 - interim WHO solidarity trial results. N Engl J Med. 2021;384(6):497-511.[27]
3. RECOVERY Collaborative Group. Lopinavir–ritonavir in patients admitted to hospital with COVID-19 (RECOVERY): a randomised, controlled, open-label, platform trial. Lancet 2020;396(10259): 1345-52.[68]

Fonte: adaptada de Bhimraj A, Morgan RL, Shumaker AH, Lavergne V, Baden L, Cheng VC, Edwards KM, Gandhi R, Gallagher J, Muller WJ, O'Horo JC, Shoham S, Murad MH, Mustafa RA, Sultan S, Falck-Ytter Y. Infectious Diseases Society of America. Guidelines on the Treatment and Management of Patients with COVID-19: Infectious Diseases Society of America; 2021; Version 5.6.0. [cited 2021 May 10]. Available from: https://www.idsociety.org/practice-guideline/covid-19-guideline-treatment-and-management/[5]

REFERÊNCIAS

1. Lotfi T, Stevens A, Akl EA, Falavigna M, Kredo T, Mathew JL, Schünemann HJ; eCOVID Collaborators. Getting trustworthy guidelines into the hands of decision-makers and supporting their consideration of contextual factors for implementation globally: recommendation mapping of COVID-19 guidelines. J Clin Epidemiol. 2021;135:182-6.
2. COVID-19 Recommendations - RecMap. 2021. COVID19 Recommendations and Gateway to Contextualization. [cited 2021 May 10].Available from: https://covid19.recmap.org
3. The WHO Rapid Evidence Appraisal for COVID-19 Therapies Working Group, Sterne JA, Murthy S, Diaz JV, Slutsky AS, Villar J, Angus DC, et al. Association Between Administration of Systemic Corticosteroids and Mortality Among Critically Ill Patients with COVID-19: A Meta-analysis. JAMA. 2020;324(13):1330-41.
4. RECOVERY Collaborative Group, Horby P, Lim WS, Emberson JR, Mafham M, Bell JL, Linsell L, et al. Dexamethasone in Hospitalized Patients with Covid-19. N Engl J Med. 2021;384(8):693-704.
5. Bhimraj A, Morgan RL, Shumaker AH, Lavergne V, Baden L, Cheng VC, Edwards KM, Gandhi R, Gallagher J, Muller WJ, O'Horo JC, Shoham S, Murad MH, Mustafa RA, Sultan S, Falck-Ytter Y. Infectious Diseases Society of America. Guidelines on the Treatment and Management of Patients with COVID-19: Infectious Diseases Society of America; 2021; Version 5.6.0. [cited 2021 May 10]. Available from: https://www.idsociety.org/practice-guideline/covid-19-guideline-treatment-and-management/
6. Lemos AC, do Espírito Santo DA, Salvetti MC, Gilio RN, Agra LB, Pazin-Filho A, et al. Therapeutic versus prophylactic anticoagulation for severe COVID-19: a randomized phase II clinical trial (HESACOVID). Thromb Res. 2020;196:359-66.
7. INSPIRATION Investigators, Sadeghipour P, Talasaz AH, Rashidi F, Sharif-Kashani B, Beigmohammadi MT, Farrokhpour M, et al. Effect of intermediate-dose vs standard-dose prophylactic anticoagulation on thrombotic events, extracorporeal membrane oxygenation treatment, or mortality among patients with COVID-19 admitted to the intensive care unit: the INSPIRATION randomized clinical trial. JAMA. 2021;325(16):1620-30.
8. Perepu US, Chambers I, Wahab A, Ten Eyck P, Wu C, Dayal S, et al. Standard prophylactic versus intermediate dose enoxaparin in adults with severe COVID-19: a multi-center, open-label, randomized controlled trial. J Thromb Haemost. 2021;19(9):2225-34.
9. REMAP-CAP Investigators; ACTIV-4a Investigators; ATTACC Investigators, Goligher EC, Bradbury CA, McVerry BJ, et al. Therapeutic anticoagulation with heparin in critically ill patients with Covid-19. N Engl J Med. 2021;385(9):777-89.
10. Spyropoulos AC, Goldin M, Giannis D, Diab W, Wang J, Khanijo S, Mignatti A, Gianos E, Cohen M, Sharifova G, Lund JM, Tafur A, Lewis PA, Cohoon KP, Rahman H, Sison CP, Lesser ML, Ochani K, Agrawal N, Hsia J, Anderson VE, Bonaca M, Halperin JL, Weitz JI; HEP-COVID Investigators. Efficacy and safety of therapeutic-dose heparin vs standard prophylactic or intermediate-dose heparins for thromboprophylaxis in high-risk hospitalized patients with COVID-19: the HEP-COVID randomized clinical trial. JAMA Intern Med. 2021 Oct 7:e216203.
11. National COVID-19 Clinical Evidence Taskforce. Caring for people with COVID-19. Supporting Australia's healthcare professionals with continually updated, evidence-based clinical guidelines. 2021. [cited 2021 May10]. Available from: https://covid19evidence.net.au/#living-guidelines
12. Sholzberg M, Tang GH, Rahhal H, AlHamzah M, Kreuziger LB, Áinle FN, Alomran F, Alayed K, Alsheef M, AlSumait F, Pompilio CE, Sperlich C, Tangri S, Tang T, Jaksa P, Suryanarayan D, Almarshoodi M, Castellucci LA, James PD, Lillicrap D, Carrier M, Beckett A, Colovos C, Jayakar J, Arsenault MP, Wu C, Doyon K, Andreou ER, Dounaevskaia V, Tseng EK, Lim G, Fralick M, Middeldorp S, Lee AYY, Zuo F, da Costa BR, Thorpe KE, Negri EM, Cushman M, Jüni P; RAPID trial investigators. Effectiveness of therapeutic heparin versus prophylactic heparin on death, mechanical ventilation, or intensive care unit admission in moderately ill patients with covid-19 admitted to hospital: RAPID randomised clinical trial. BMJ. 2021;375:n2400.
13. Lopes RD, de Barros e Silva PG, Furtado RH, Macedo AV, Bronhara B, Damiani LP, Barbosa LM, de Aveiro Morata J, Ramacciotti E, de Aquino Martins P, de Oliveira AL, Nunes VS, Ritt LE, Rocha AT, Tramujas L, Santos SV, Diaz DR, Viana LS, Melro LM, de Alcântara Chaud MS, Figueiredo EL, Neuenschwander FC, Dracoulakis MD, Lima RG, de Souza Dantas VC, Fernandes AC, Gebara OC, Hernandes ME, Queiroz DA, Veiga VC, Canesin MF, de Faria LM, Feitosa-Filho GS, Gazzana MB, Liporace IL, de Oliveira Twardowsky A, Maia LN, Machado FR, de Matos Soeiro A, Conceição-Souza GE, Armaganijan L, Guimarães PO, Rosa RG, Azevedo LC, Alexander JH, Avezum A, Cavalcanti AB, Berwanger O; ACTION Coalition COVID-19 Brazil IV Investigators. Therapeutic versus prophylactic anticoagulation for patients admitted to hospital with COVID-19 and elevated D-dimer concentration (ACTION): an open-label, multicentre, randomised, controlled trial. Lancet. 2021;397(10291):2253-63
14. ATTACC Investigators; ACTIV-4a Investigators; REMAP-CAP Investigators, Lawler PR, Goligher EC, Berger JS, et al. Therapeutic anticoagulation with heparin in noncritically ill patients with Covid-19. N Engl J Med. 2021;385(9):790-802.
15. The REMAP-CAP Investigators, Gordon AC, Mouncey PR, Al-Beidh F, Rowan KM, Nichol AD, Arabi YM, et al. Interleukin-6 receptor antagonists in critically ill patients with Covid-19 - Preliminary report. medRxiv. 2021:2021.01.07.21249390.
16. Rosas IO, Bräu N, Waters M, Go RC, Hunter BD, Bhagani S, et al. Tocilizumab in hospitalized patients with COVID-19 pneumonia. medRxiv 2020:2020.08.27.20183442.
17. Hermine O, Mariette X, Tharaux PL, Resche-Rigon M, Porcher R, Ravaud P; CORIMUNO-19 Collaborative Group. Effect of tocilizumab vs usual care in adults hospitalized with COVID-19 and moderate or severe pneumonia: a randomized clinical trial. JAMA Intern Med. 2021;181(1):32-40.
18. Salama C, Han J, Yau L, Reiss WG, Kramer B, Neidhart JD, et al. Tocilizumab in patients hospitalized with Covid-19 pneumonia. N Engl J Med. 2021;384(1):20-30.
19. Salvarani C, Dolci G, Massari M, Merlo DF, Cavuto S, Savoldi L, Bruzzi P, Boni F, Braglia L, Turrà C, Ballerini PF, Sciascia R, Zammarchi L, Para O, Scotton PG, Inojosa WO, Ravagnani V, Salerno ND, Sainaghi PP, Brignone A, Codeluppi M, Teopompi E, Milesi M, Bertomoro P, Claudio N, Salio M, Falcone M, Cenderello G, Donghi L, Del Bono V, Colombelli PL, Angheben A, Passaro A, Secondo G, Pascale R, Piazza I, Facciolongo N, Costantini M; RCT-TCZ-COVID-19 Study Group. Effect of tocilizumab vs standard care on clinical worsening in patients hospitalized with COVID-19 pneumonia: a randomized clinical trial. JAMA Intern Med. 2021;181(1):24-31.
20. Stone JH, Frigault MJ, Serling-Boyd NJ, Fernandes AD, Harvey L, Foulkes AS, Horick NK, Healy BC, Shah R, Bensaci AM, Woolley AE, Nikiforow S, Lin N, Sagar M, Schrager H, Huckins DS, Axelrod M, Pincus MD, Fleisher J, Sacks CA, Dougan M, North CM, Halvorsen YD, Thurber TK, Dagher Z, Scherer A, Wallwork RS, Kim AY, Schoenfeld S, Sen P, Neilan TG, Perugino CA, Unizony SH, Collier DS, Matza MA, Yinh JM, Bowman KA, Meyerowitz E, Zafar A, Drobni ZD, Bolster MB, Kohler M, D'Silva KM, Dau J, Lockwood MM, Cubbison C, Weber BN, Mansour MK; BACC Bay Tocilizumab Trial Investigators. Efficacy of tocilizumab in patients hospitalized with Covid-19. N Engl J Med. 2020;383(24):2333-44.
21. Veiga VC, Prats JA, Farias DL, Rosa RG, Dourado LK, Zampieri FG, Machado FR, Lopes RD, Berwanger O, Azevedo LC, Avezum Á, Lisboa TC, Rojas SS, Coelho JC, Leite RT, Carvalho JC, Andrade LE, Sandes AF, Pintão MC, Castro CG Jr, Santos SV, de Almeida TM, Costa AN, Gebara OC, de Freitas FG, Pacheco ES, Machado DJ, Martin J, Conceição FG, Siqueira SR, Damiani LP, Ishihara LM, Schneider D, de Souza D, Cavalcanti AB, Scheinberg P; Coalition covid-19 Brazil VI Investigators. Effect of tocilizumab on clinical outcomes at 15 days in patients with severe or critical coronavirus disease 2019: randomised controlled trial. BMJ. 2021;372:n84.
22. RECOVERY Collaborative Group. Tocilizumab in patients admitted to hospital with COVID-19 (RECOVERY): a randomised, controlled, open-label, platform trial. Lancet. 2021;397(10285):1637-45.

23. RECOVERY Collaborative Group, Horby PW, Pessoa-Amorim G, Peto L, Brightling CE, Sarkar R, Thomas K, et al. Tocilizumab in patients admitted to hospital with COVID-19 (RECOVERY): preliminary results of a randomised, controlled, open-label, platform trial. medRxiv. 2021:2021.02.11.21249258.
24. REMAP-CAP Investigators, Gordon AC, Mouncey PR, Al-Beidh F, Rowan KM, Nichol AD, Arabi YM, et al. Interleukin-6 receptor antagonists in critically ill patients with Covid-19. N Engl J Med. 2021;384(16):1491-502.
25. RECOVERY Collaborative Group, Horby P, Mafham M, Linsell L, Bell JL, Staplin N, Emberson JR, et al. Effect of hydroxychloroquine in hospitalized patients with COVID-19. N Engl J Med. 2020;383(21):2030-40.
26. Cavalcanti AB, Zampieri FG, Rosa RG, Azevedo LC, Veiga VC, Avezum A, Damiani LP, Marcadenti A, Kawano-Dourado L, Lisboa T, Junqueira DL, de Barros E Silva PG, Tramujas L, Abreu-Silva EO, Laranjeira LN, Soares AT, Echenique LS, Pereira AJ, Freitas FG, Gebara OC, Dantas VC, Furtado RH, Milan EP, Golin NA, Cardoso FF, Maia IS, Hoffmann Filho CR, Kormann AP, Amazonas RB, Bocchi de Oliveira MF, Serpa-Neto A, Falavigna M, Lopes RD, Machado FR, Berwanger O; Coalition Covid-19 Brazil I Investigators. Hydroxychloroquine with or without azithromycin in mild-to-moderate Covid-19. N Engl J Med. 2020;383(21):2041-52.
27. WHO Solidarity Trial Consortium, Pan H, Peto R, Henao-Restrepo AM, Preziosi MP, Sathiyamoorthy V, Abdool Karim Q, et al. Repurposed antiviral drugs for Covid-19 - interim WHO solidarity trial results. N Engl J Med. 2021;384(6):497-511.
28. Self WH, Semler MW, Leither LM, Casey JD, Angus DC, Brower RG, et al. Effect of hydroxychloroquine on clinical status at 14 days in hospitalized patients with COVID-19: a randomized clinical trial. JAMA. 2020;324(21):2165-76.
29. Ulrich RJ, Troxel AB, Carmody E, Eapen J, Bäcker M, DeHovitz JA, et al. Treating COVID-19 with hydroxychloroquine (TEACH): a multicenter, double-blind randomized controlled trial in hospitalized patients. Open Forum Infect Dis. 2020;7(10):ofaa446
30. Rosenberg ES, Dufort EM, Udo T, Wilberschied LA, Kumar J, Tesoriero J, et al. Association of treatment with hydroxychloroquine or azithromycin with in-hospital mortality in patients with COVID-19 in New York State. JAMA. 2020;323(24):2493-502.
31. Chen J, Liu D, Liu L, Liu P, Xu Q, Xia L, et al. [A pilot study of hydroxychloroquine in treatment of patients with moderate COVID-19]. Zhejiang Da Xue Xue Bao Yi Xue Ban. 2020;49(2):215-9. Chinese.
32. Chen Z, Hu J, Zhang Z, Jiang S, Han S, Yan D, et al. Efficacy of hydroxychloroquine in patients with COVID-19: results of a randomized clinical trial. medRxiv 2020:2020.03.22.20040758.
33. Tang W, Cao Z, Han M, Wang Z, Chen J, Sun W, et al. Hydroxychloroquine in patients with mainly mild to moderate coronavirus disease 2019: open label, randomised controlled trial. BMJ. 2020;369:m1849.
34. Mahévas M, Tran VT, Roumier M, Chabrol A, Paule R, Guillaud C, et al. No evidence of clinical efficacy of hydroxychloroquine in patients hospitalised for COVID-19 infection and requiring oxygen: results of a study using routinely collected data to emulate a target trial. medRxiv 2020:2020.04.10.20060699.
35. Magagnoli J, Narendran S, Pereira F, Cummings TH, Hardin JW, Sutton SS, et al. Outcomes of hydroxychloroquine usage in United States veterans hospitalized with Covid-19. Med (N Y). 2020;1(1):114-127.e3.
36. Ip A, Berry DA, Hansen E, Goy AH, Pecora AL, Sinclaire BA, et al. Hydroxychloroquine and tocilizumab therapy in COVID-19 patients-An observational study. PLoS One. 2020;15(8):e0237693.
37. Gautret P, Lagier JC, Parola P, Hoang VT, Meddeb L, Mailhe M, et al. Hydroxychloroquine and azithromycin as a treatment of COVID-19: results of an open-label non-randomized clinical trial. Int J Antimicrob Agents. 2020;56(1):105949.
38. Gautret P, Lagier JC, Parola P, et al. Clinical and microbiological effect of a combination of hydroxychloroquine and azithromycin in 80 COVID-19 patients with at least a six-day follow up: a pilot observational study. Travel Med Infect Dis. 2020;34:101663.
39. Molina JM, Delaugerre C, Le Goff J, Mela-Lima B, Ponscarme D, Goldwirt L, et al. No evidence of rapid antiviral clearance or clinical benefit with the combination of hydroxychloroquine and azithromycin in patients with severe COVID-19 infection. Med Mal Infect. 2020;50(4):384.
40. Chorin E, Dai M, Shulman E, Wadhwani L, Roi-Bar-Cohen, Barbhaiya C, et al. The QT interval in patients with SARS-CoV-2 infection treated with hydroxychloroquine/azithromycin. medRxiv. 2020:2020.04.02.20047050.
41. Wang Y, Zhang D, Du G, Du R, Zhao J, Jin Y, et al. Remdesivir in adults with severe COVID-19: a randomised, double-blind, placebo-controlled, multicentre trial. Lancet. 2020;395(10236):1569-78.[41]
42. Beigel JH, Tomashek KM, Dodd LE, Mehta AK, Zingman BS, Kalil AC, Hohmann E, Chu HY, Luetkemeyer A, Kline S, Lopez de Castilla D, Finberg RW, Dierberg K, Tapson V, Hsieh L, Patterson TF, Paredes R, Sweeney DA, Short WR, Touloumi G, Lye DC, Ohmagari N, Oh MD, Ruiz-Palacios GM, Benfield T, Fätkenheuer G, Kortepeter MG, Atmar RL, Creech CB, Lundgren J, Babiker AG, Pett S, Neaton JD, Burgess TH, Bonnett T, Green M, Makowski M, Osinusi A, Nayak S, Lane HC; ACTT-1 Study Group Members. Remdesivir for the treatment of Covid-19 - Final report. N Engl J Med. 2020;383(19):1813-26.
43. Spinner CD, Gottlieb RL, Criner GJ, et al. Effect of Remdesivir vs Standard Care on Clinical Status at 11 Days in Patients with Moderate COVID-19: A Randomized Clinical Trial. JAMA 2020;324(11):1048-57.
44. Li L, Zhang W, Hu Y, Tong X, Zheng S, Yang J, et al. Effect of convalescent plasma therapy on time to clinical improvement in patients with severe and life-threatening COVID-19: a randomized clinical trial. JAMA. 2020;324(5):460-70.
45. Gharbharan A, Jordans CC, Geurtsvankessel C, den Hollander JG, Karim F, Mollema FP, et al. Convalescent plasma for COVID-19. A randomized clinical trial. medRxiv. 2020:2020.07.01.20139857.
46. AlQahtani M, Abdulrahman A, Almadani A, Alali SY, Al Zamrooni AM, Hejab AH, et al. Randomized controlled trial of convalescent plasma therapy against standard therapy in patients with severe COVID-19 disease. Sci Rep. 2021;11(1):9927.
47. Avendaño-Solà C, Ramos-Martínez A, Muñez-Rubio E, Ruiz-Antorán B, de Molina RM, Torres F, et al. Convalescent plasma for COVID-19: a multicenter, randomized clinical trial. medRxiv. 2020:2020.08.26.20182444.
48. Ray Y, Paul SR, Bandopadhyay P, D'Rozario R, Sarif J, Lahiri A, et al. Clinical and immunological benefits of convalescent plasma therapy in severe COVID-19: insights from a single center open label randomised control trial. medRxiv 2020.11.25.20237883.[48]
49. Simonovich VA, Burgos Pratx LD, Scibona P, Beruto MV, Vallone MG, Vázquez C, et al. A randomized trial of convalescent plasma in Covid-19 severe pneumonia. N Engl J Med. 2021;384(7):619-29.
50. Agarwal A, Mukherjee A, Kumar G, Chatterjee P, Bhatnagar T, Malhotra P; PLACID Trial Collaborators. Convalescent plasma in the management of moderate covid-19 in adults in India: open label phase II multicentre randomised controlled trial (PLACID Trial). BMJ. 2020;371:m3939.
51. O'Donnell MR, Grinsztejn B, Cummings MJ, Justman JE, Lamb MR, Eckhardt CM, et al. A randomized, double-blind controlled trial of convalescent plasma in adults with severe COVID-19. J Clin Invest. 2021;131(13):e150646.
52. RECOVERY Collaborative Group. Convalescent plasma in patients admitted to hospital with COVID-19 (RECOVERY): a randomised, controlled, open-label, platform trial. Lancet. 2021;397(10289):2049-59.

53. Balcells ME, Rojas L, Le Corre N, Martínez-Valdebenito C, Ceballos ME, Ferrés M, et al. Early versus deferred anti-SARS-CoV-2 convalescent plasma in patients admitted for COVID-19: a randomized phase II clinical trial. PLoS Med. 2021;18(3):e1003415.

54. Joyner MJ, Bruno KA, Klassen SA, Kunze KL, Johnson PW, Lesser ER, et al. Safety update: COVID-19 convalescent plasma in 20,000 hospitalized patients. Mayo Clin Proc. 2020;95(9):1888-97.

55. Libster R, Pérez Marc GP, Wappner D, Coviello S, Bianchi A, Braem V, et al. Prevention of severe COVID-19 in the elderly by early high-titer plasma. medRxiv. 2020:2020.11.20.20234013.

56. Gorial FI, Mashhadani S, Sayaly HM, Dakhil BD, AlMashhadani MM, Aljabory AM, et al. Effectiveness of Ivermectin as add-on Therapy in COVID-19 Management (Pilot Trial). medRxiv 2020:2020.07.07.20145979.

57. Hashim HA, Maulood MF, Rasheed AM, Fatak DF, Kabah KK, Abdulamir AS. Controlled randomized clinical trial on using ivermectin with doxycycline for treating COVID-19 patients in Baghdad, Iraq. medRxiv 2020:2020.10.26.20219345.

58. Rajter JC, Sherman MS, Fatteh N, Vogel F, Sacks J, Rajter JJ. Use of ivermectin is associated with lower mortality in hospitalized patients with coronavirus disease 2019: the ivermectin in COVID nineteen study. Chest. 2021;159(1):85-92.

59. Chaccour C, Casellas A, Blanco-Di Matteo A, Pineda I, Fernandez-Montero A, Ruiz-Castillo P, et al. The effect of early treatment with ivermectin on viral load, symptoms and humoral response in patients with non-severe COVID-19: a pilot, double-blind, placebo-controlled, randomized clinical trial. EClinicalMedicine. 2021;32:100720.

60. Chachar AZ, Khan KA, Asif M, Khushbakht, Khaqan A, Basri R. Effectiveness of ivermectin in SARS-CoV-2/COVID-19 patients. Int J Sci. 2020;9:31-5.

61. Ahmed S, Karim MM, Ross AG, Hossain MS, Clemens JD, Sumiya MK, et al. A five-day course of ivermectin for the treatment of COVID-19 may reduce the duration of illness. Int J Infect Dis. 2021;103:214-6.

62. Podder CS, Chowdhury N, Sina MI, Haque W. Outcome of ivermectin treated mild to moderate COVID-19 cases: a single-centre, open-label, randomised controlled study. IMC J Med Sci. 2020;14(2):002.

63. Deftereos SG, Giannopoulos G, Vrachatis DA, Siasos GD, Giotaki SG, Gargalianos P, Metallidis S, Sianos G, Baltagiannis S, Panagopoulos P, Dolianitis K, Randou E, Syrigos K, Kotanidou A, Koulouris NG, Milionis H, Sipsas N, Gogos C, Tsoukalas G, Olympios CD, Tsagalou E, Migdalis I, Gerakari S, Angelidis C, Alexopoulos D, Davlouros P, Hahalis G, Kanonidis I, Katritsis D, Kolettis T, Manolis AS, Michalis L, Naka KK, Pyrgakis VN, Toutouzas KP, Triposkiadis F, Tsioufis K, Vavouranakis E, Martinèz-Dolz L, Reimers B, Stefanini GG, Cleman M, Goudevenos J, Tsiodras S, Tousoulis D, Iliodromitis E, Mehran R, Dangas G, Stefanadis C; GRECCO-19 investigators. Effect of colchicine vs standard care on cardiac and inflammatory biomarkers and clinical outcomes in patients hospitalized with coronavirus disease 2019: the GRECCO-19 randomized clinical trial. JAMA Netw Open. 2020;3(6):e2013136.

64. Lopes MI, Bonjorno LP, Giannini MC, Amaral NB, Menezes PI, Dib SM, et al. Beneficial effects of colchicine for moderate to severe COVID-19: a randomised, double-blinded, placebo controlled clinical trial. RMD Open. 2021;7(1):e001455.

65. RECOVERY Collaborative Group. Colchicine in patients admitted to hospital with COVID-19 (RECOVERY): a randomised, controlled, open-label, platform trial. Lancet Respir Med. 2021;9(12):1419-26.

66. Chalmers JD, Crichton ML, Goeminne PC, Cao B, Humbert M, Shteinberg M, et al. Management of hospitalised adults with coronavirus disease-19 (COVID-19): A European Respiratory Society living guideline. Eur Respir J. 2021;57(4):2100048.

67. Cao B, Wang Y, Wen D, Liu W, Wang J, Fan G, et al. A trial of lopinavir-ritonavir in adults hospitalized with severe Covid-19. N Engl J Med. 2020;382(19):1787-99.

68. RECOVERY Collaborative Group. Lopinavir-ritonavir in patients admitted to hospital with COVID-19 (RECOVERY): a randomised, controlled, open-label, platform trial. Lancet. 2020;396(10259):1345-52.
